# Role of the Transcription Factor Yin Yang 1 and Its Selectively Identified Target Survivin in High-Grade B-Cells Non-Hodgkin Lymphomas: Potential Diagnostic and Therapeutic Targets

**DOI:** 10.3390/ijms21176446

**Published:** 2020-09-03

**Authors:** Silvia Vivarelli, Luca Falzone, Giovanni Ligresti, Saverio Candido, Adriana Garozzo, Gaetano Giuseppe Magro, Benjamin Bonavida, Massimo Libra

**Affiliations:** 1 Laboratory of Translational Oncology, Department of Biomedical and Biotechnological Sciences, University of Catania, 95123 Catania, Italy; silvia.vivarelli7@gmail.com (S.V.); ligresti@bu.edu (G.L.); scandido@unict.it (S.C.); 2 Epidemiology Unit, IRCCS Istituto Nazionale Tumori ‘Fondazione G. Pascale’, 80131 Naples, Italy; l.falzone@istitutotumori.na.it; 3 Research Center for Prevention, Diagnosis and Treatment of Cancer, University of Catania, 95123 Catania, Italy; agar@unict.it (A.G.); g.magro@unict.it (G.G.M.); 4 Laboratory of Virology, Department of Biomedical and Biotechnological Sciences, University of Catania, 95123 Catania, Italy; 5 Department of Medical and Surgical Sciences and Advanced Technology “G.F. Ingrassia”, University of Catania, 95123 Catania, Sicily, Italy; 6 Department of Microbiology, Immunology and Molecular Genetics, David Geffen School of Medicine, University of California, Los Angeles, CA 90095, USA; bbonavida@mednet.ucla.edu

**Keywords:** Yin Yang 1, *BIRC5*/survivin, B-cell non-Hodgkin lymphoma, Burkitt’s lymphoma, chemotherapy, apoptosis

## Abstract

B-cell non-Hodgkin lymphomas (B-NHLs) are often characterized by the development of resistance to chemotherapeutic drugs and/or relapse. During drug-induced apoptosis, Yin Yang 1 (*YY1*) transcription factor might modulate the expression of apoptotic regulators genes. The present study was aimed to: (1) examine the potential oncogenic role of *YY1* in reversing drug resistance in B-NHLs; and (2) identify *YY1* transcriptional target(s) that regulate the apoptotic pathway in B-NHLs. Predictive analyses coupled with database-deposited data suggested that *YY1* binds the promoter of the *BIRC5*/survivin anti-apoptotic gene. Gene Expression Omnibus (GEO) analyses of several B-NHL repositories revealed a conserved positive correlation between *YY1* and survivin, both highly expressed, especially in aggressive B-NHLs. Further validation experiments performed in Raji Burkitt’s lymphomas cells, demonstrated that *YY1* silencing was associated with survivin downregulation and sensitized the cells to apoptosis. Overall, our results revealed that: (1) *YY1* and survivin are positively correlated and overexpressed in B-NHLs, especially in BLs; (2) *YY1* strongly binds to the survivin promoter, hence survivin may be suggested as *YY1* transcriptional target; (3) *YY1* silencing sensitizes Raji cells to drug-induced apoptosis via downregulation of survivin; (4) both *YY1* and survivin are potential diagnostic markers and therapeutic targets for the treatment of resistant/relapsed B-NHLs.

## 1. Introduction

Worldwide, non-Hodgkin lymphomas (NHLs) represent the tenth most frequent cancers in males and the twelfth most diffused tumors in females, respectively, with an estimation of 509,590 new cases and 248,724 deaths in the past 2018, according to The Global Cancer Observatory (GLOBOCAN) of the World Health Organization (WHO) [1]. This group of tumors stems from the malignant transformation of mature and immature cells of the immune system, affecting either B lymphocytes (B cells, representing around 86% of all NHLs), but also T- and natural killer (NK) cells (14% of all NHLs) [1]. According to the disease progression features, B-cells NHLs (B-NHLs) are further divided in aggressive—fast progressing—including Diffuse large B-cell lymphoma (DLBCL) and Burkitt’s lymphoma (BL) and indolent—slow growing—such as for example Follicular lymphoma (FL), Mantle Cell lymphoma (MCL), B-cell small lymphocytic lymphoma/chronic lymphocytic leukemia, Marginal Zone B-cell lymphoma (MZL) and lymphoplasmacytic lymphoma/Waldenström macroglobulinemia [2]. The main issue with NHLs, which could explain the still high global mortality rate, consists in their frequent resistance following anti-cancer treatments and relapse, which the current research is trying to fight by developing novel therapeutic strategies [3,4].

Yin Yang 1 (*YY1*) is a C_2_H_2_-type zinc finger protein, conserved amongst species and found ubiquitously expressed in the majority of human tissues and organs. This transcription factor is able to control, either activating or repressing, the transcription of up to the 7% of the whole human genome [5]. For that reason, *YY1* may exert profound effects on several important cellular pathways, including the control of cell cycle, DNA repair, chromatin recruitment of Polycomb Group (PcG) proteins, chromatin remodeling, modulation of cellular metabolism, cell survival and programmed cell death [6]. Importantly, the molecular mechanisms by which *YY1* modulates the transcription of its target genes are very heterogeneous and strictly dependent on the cellular-specific context [7,8,9]. In fact, *YY1* positively or negatively regulates the expression of target genes directly, by binding a conserved 12-mer consensus sequence located within the DNA transcriptional regulatory region, or indirectly, following the interaction either with other transcription factors or with co-activators/co-repressors of the transcription, as well as general epigenetic modulators [6].

In cancer, the pleiotropic *YY1* transcription factor plays a controversial role. It is still unclear in which cases *YY1* acts as an oncogene or as a tumor suppressor. Therefore, a better comprehension of the *YY1*-mediated molecular mechanisms that are activated or inhibited in the different kind of cancers may help in the development of novel diagnostics, as well as effective therapeutic strategies [9].

Notably, *YY1* plays a crucial role at all stages of B-cells development, in the immunoglobulin class switch recombination process and, also, during lymphomagenesis [10]. Prevalently, in hematological malignancies the role of *YY1* seems to be pro-tumorigenic [11]. In this regard, our laboratory has previously demonstrated that *YY1* is significantly overexpressed in high-grade lymphomas, including BLs and DLBCLs, when compared to normal B cells [12].

Although previous reports highlighted that *YY1* inhibition resulted in the increased sensitization of NHL cells to drug- or immune- induced cell death, all downstream pathways modulated by *YY1* have not been comprehensively characterized yet [13,14]. 

The goal of this study was to better understand the oncogenic function played by *YY1* in the regulation of the apoptotic response following chemotherapeutic treatments, and to further shed light on the possible downstream targets of this transcription factor. In-silico JASPAR binding prediction, corroborated by in vitro *YY1*-ChIP-seq experiments deposited in ENCODE, demonstrated that *YY1* strongly binds the *BIRC5*/survivin promoter, which is a negative regulator of the apoptosis. In addition, the computational analysis performed on several B-NHLs Gene Expression Omnibus (GEO) curated gene expression datasets gave new insights on the significantly correlated upregulation of both *YY1* and survivin in cancer patients compared to normal subjects. Importantly, the positive correlation between *YY1* and survivin expression was present in all the B-NHLs datasets analyzed, and it was consistently higher in aggressive B-NHLs specimens, including BLs.

Subsequently, by using a cellular model of—aggressive—BL, the Raji cell line, the roles of *YY1* and survivin were further validated. Through a shRNA-mediated silencing approach it was possible to assess that survivin was selectively downregulated in association with *YY1* knock-down, thus confirming that *YY1* may be a potential positive transcriptional regulator of survivin in Raji BL cells. Moreover, the effect of modulating *YY1* expression levels on Raji cellular growth, as well as on cellular viability following anti-cancer treatments was evaluated, confirming the pro-tumorigenic role of both *YY1* and survivin in these cells. Overall, our findings suggest a potential diagnostic, as well as therapeutic role for both *YY1* and survivin in B-NHLs.

## 2. Results

### 2.1. JASPAR Screening Allows the Identification of YY1 Putative Binding Sites on the Transcriptional Regulatory Regions of Several Apoptotic Genes: Identification of BIRC5/Survivin

During both B-NHL genesis and progression, *YY1* mainly plays a pro-tumorigenic role. Recent studies suggested that *YY1* negatively regulates apoptosis in B-NHL cells, therefore promoting pro-survival programs and, in turn, resistance to cytotoxic stimuli. To identify the potential direct transcriptional targets of *YY1* in B-NHLs, JASPAR in-silico analysis was performed to search for the presence of *YY1* putative binding sites located within the transcriptional regulatory region of the main genes involved in the modulation of the apoptosis, including the BCL2 family members, as well as IAPs members. 

Once the 3000 bp long transcriptional regulatory sequence for each considered gene was identified through the use of Ensembl, the analysis of each sequence has been pursued with JASPAR open-access database, by using the deposited matrix of 12-mer binding domain for *YY1*. Following the in-silico analysis, a panel of 16 best putative *YY1* targets candidates was shortlisted. The features of such selected genes in terms of the overall number of *YY1* putative binding sites and the homology score have been reported in Table S1. All of the genes reported in the Table show more than one putative binding site for *YY1* in their transcriptional regulatory region, with an homology higher than 80% with the 12-mer *YY1*-matrix sequence deposited in JASPAR, thus suggesting that all of them may be potentially bound by *YY1* within their regulatory regions (Table S1). Amongst the best candidates, *BIRC5* (or survivin), a member of the IAPs, was selected for further characterization, as it carries 5 putative *YY1*-binding sites along its DNA transcriptional regulatory region, with a homology higher than 80% (Table 1).

### 2.2. YY1-ChIP-Sequencing Data Confirm That YY1 Strongly Binds the BIRC5 Promoter

Bioinformatics analysis of “Transcription Factor ChIP-seq” experiments performed through the UCSC Genome Browser (Homo sapiens genome assembly GRCh38), revealed that the promoter region of *BIRC5* (from −2000 bp upstream to +1000 downstream the TSS) is selectively bound by 138 different transcription factors, in turn able to modulate, with different strength, *BIRC5* expression (Figure S1).

**Figure 1:**
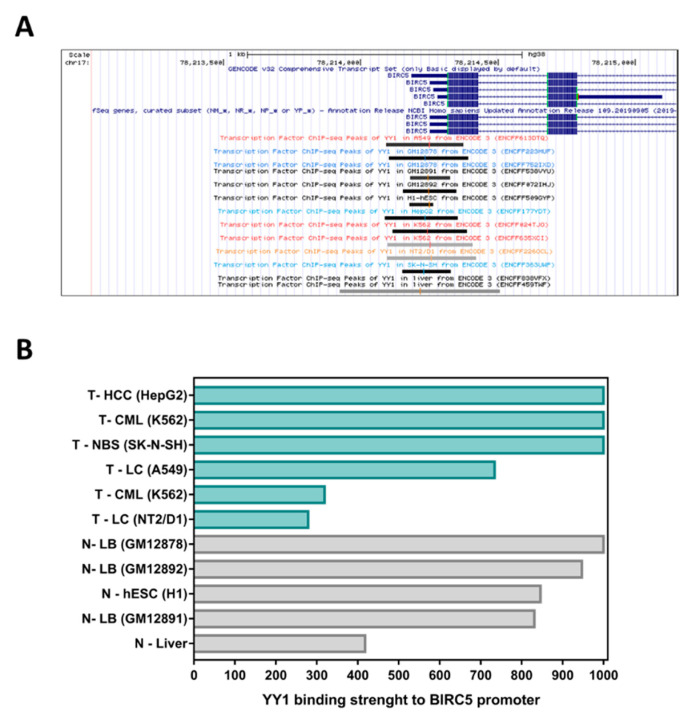
*YY1* Transcription Factor ChIP-seq binding-peaks within *BIRC5* promoter, according to public results available in ENCODE. (**A**). Black and grey boxes identify peaks of *YY1* occupancy within the *BIRC5* promoter (from −2000 to +1000 bp around the TSS). The length of each box indicates the region of binding within the promoter, while the gray-scale of the boxes is proportional to the strength of the binding of *YY1* transcription factor to *BIRC5* promoter. (**B**). Bar plot summarizing the binding strength of *YY1* binding to *BIRC5* promoter found in each positive experiment (signal strength score ranges from 0 to 1000). T = tumor samples (green); N = normal samples (grey).

Amongst these transcription factors, *YY1* showed moderate to high interaction strength with the *BIRC5* promoter within 11 out of 13 *YY1*-ChIP-Seq experiments performed on different specimens, including three different lymphoid cell lines (respectively: GM12878, GM12891 and GM12892; Figure 1A). It is important to note that *YY1* binds the *BIRC5* promoter in both cancer and normal cell lines. However, by comparing the binding intensity obtained for *YY1* binding to the *BIRC5* promoter in normal liver tissues vs. hepatocellular carcinoma, it is possible to observe that *YY1* shows a stronger interaction with the *BIRC5* promoter in cancer tissues than in normal ones (i.e., strength of 418 in normal cells vs. 1000 in cancer cells; Figure 1B and Table S2).

### 2.3. Bioinformatics Data Confirm That YY1 and BIRC5 Are Positively Correlated within the B-NHL GEO Datasets and They Are Both Associated with B-NHL Clinical-Pathological Features

To validate the association between *YY1* and its hereby-identified transcriptional target *BIRC5*, several independent whole human genome expression array datasets, 12 generated with human B-NHL samples and five with normal healthy human control samples, have been selected from the National Center for Biotechnology Information GEO records (Table 2).

**Figure 2:**
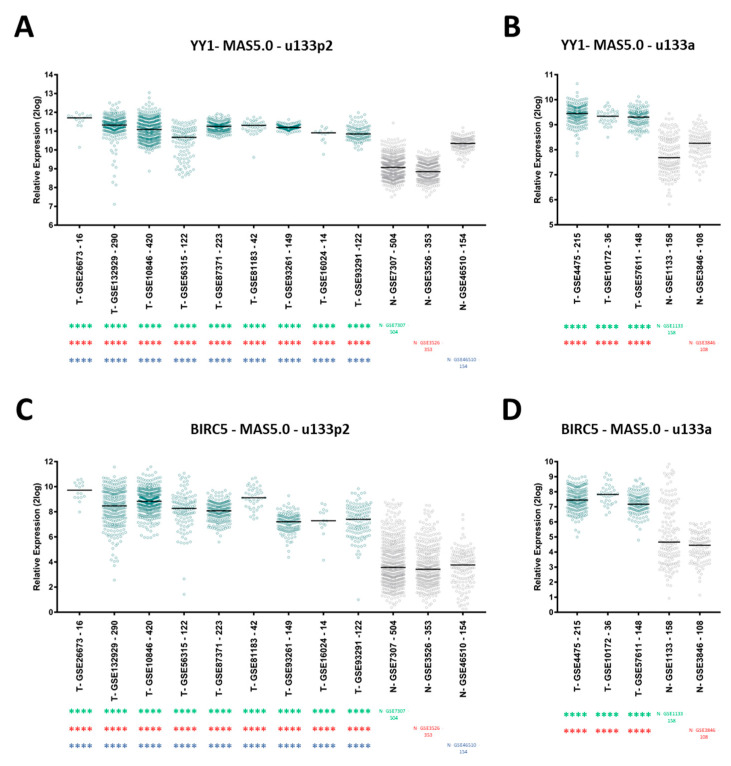
R2 analysis of *YY1* and *BIRC5* expression levels in B-NHL patients vs. healthy controls. Comparisons have been performed between datasets generated with same array and same normalization method (MAS5.0). (**A**). *YY1* relative expression levels are significantly higher in nine different B-NHL patients GEO datasets (T, tumor) compared to each of three different normal samples GEO datasets (N, Normal; u133p2 array); (**B**). *YY1* relative expression levels are significantly higher in three different B-NHL patients GEO datasets (T) compared to each of two different normal samples GEO datasets (N; u133a array); (**C**). *BIRC5* relative expression levels are significantly higher in nine different B-NHL patients GEO datasets (T) compared to each of three different normal samples GEO datasets (N; u133p2 array); (**D**). *BIRC5* relative expression levels are significantly higher in three different B-NHL patients GEO datasets (T) compared to each of two different normal samples GEO datasets (N; u133a array). The relative gene expression levels are expressed as log2 (base 2 logarithmic values). The number of samples per each dataset is indicated along with the GSE dataset ID. The results are presented as Median ± SD. One-way ANOVA with Tukey’s post-hoc test analysis has been performed (**** *p* < 0.0001).

Firstly, to investigate the expression levels of both *YY1* and *BIRC5* across the selected GEO datasets, the “megasampler module” of the R2 genomics analysis and visualization platform was used. As shown in Figure 2, the results of the R2 megasampler analysis revealed that in all the independent GEO datasets analyzed, both *YY1* and *BIRC5* are significantly upregulated within the B-NHL patients datasets (T, Tumor) compared to healthy control ones (N, Normal; *p* < 0.0001; the complete statistics results are included in Table S3), thereby suggesting that the evaluation of the expression of these two genes may have a diagnostic value in suspicious cases or in individuals at risk for this pathology.

Subsequently, both *YY1* and *BIRC5* normalized expression values distribution in the 12 B-NHLs GEO datasets have been tested for normality (D’Agostino & Pearson normality test). A summary of the normalized expression values distribution and their relative frequency histograms is reported in Figure S2.

Furthermore, the B-NHLs GEO datasets were analyzed by using the functions “correlate two genes” and “view a gene” of the R2 genomics analysis and visualization platform. In Table 3 are summarized the Pearson and Spearman correlation results obtained, expressed as R-values, which all show a positive and highly significant (significance expressed as P-value) correlation between the expression of *YY1* and *BIRC5*. Overall, the results suggest that the positive correlation of *YY1* and *BIRC5* expression may be a common feature of different classes of B-NHLs (Table 3 and Figure S3).

**Figure 3:**
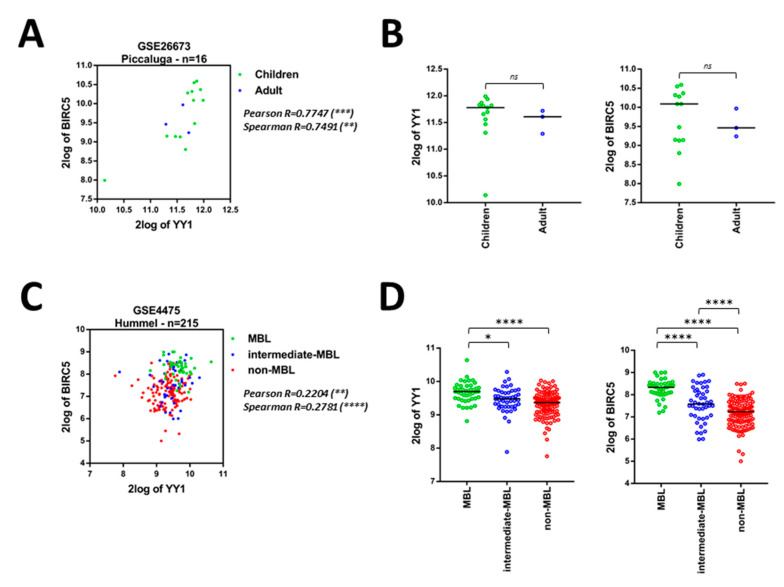

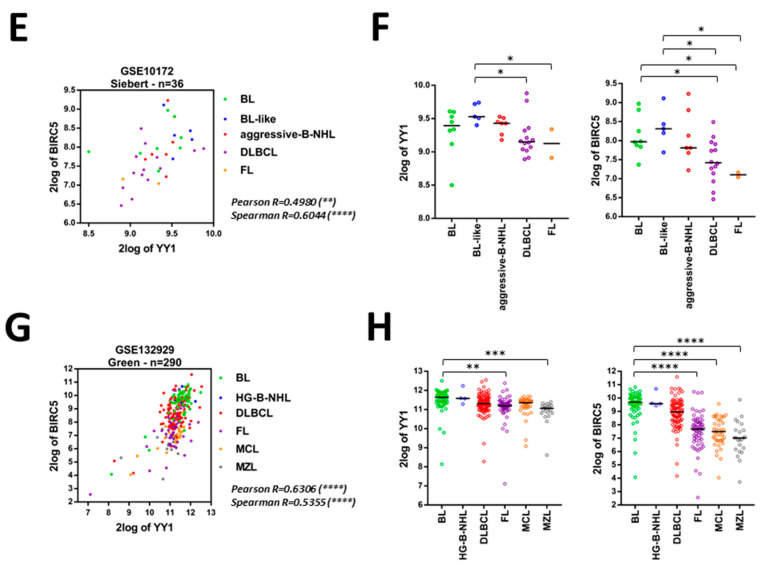
Correlation between *YY1* and *BIRC5* expression and relative *YY1* and *BIRC5* expression levels in different B-NHL subgroups, within 4 different B-NHL GEO Datasets. (**A**). GSE26673, Burkitt’s Lymphoma (BL), Pearson and Spearman correlation between *YY1* and *BIRC5* expression, patients stratified according to age (children, green; adult, blue). (**B**). GSE26673, dot plots of *YY1* (left) and *BIRC5* (right) expression with patients stratified by age (children, green; adult, blue). (**C**). GSE4475, B-NHL patients stratified based on molecular BL features (MBL green, intermediate-MBL blue, non-MBL red), Pearson and Spearman correlation between *YY1* and *BIRC5* expression. (**D**). GSE4475, dot plots of *YY1* (left) and *BIRC5* (right) expression with patients stratified based on molecular BL features (MBL green, intermediate-MBL blue, non-MBL red). (**E**). GSE10172, B-NHL patients stratified based on cancer type (BL green, BL-like blue, aggressive-B-NHL red, DLBCL purple, FL orange), Pearson and Spearman correlation between *YY1* and *BIRC5* expression. (**F**). GSE10172, dot plots of *YY1* (left) and *BIRC5* (right) expression with patients based on cancer type (BL green, BL-like blue, aggressive-B-NHL red, DLBCL purple, FL orange). (**G**). GSE132929, B-NHL patients stratified based on cancer type (BL green, HG-B-NHL blue, DLBCL red, FL purple, MCL orange, MZL grey), Pearson and Spearman correlation between *YY1* and *BIRC5* expression. (**H**). GSE132929, dot plots of *YY1* (left) and *BIRC5* (right) expression with patients based on cancer type (BL green, HG-B-NHL blue, DLBCL red, FL purple, MCL orange, MZL grey). Data in B, D, F, H are presented as dotted plots with Median ± SD. Significance was evaluated using one-way ANOVA with Tukey’s post-hoc comparison test (* *p* < 0.05; ** *p* < 0.01; *** *p* < 0.001; **** *p* < 0.0001; ns, not significant).

Importantly, four out of the 12 GEO datasets, containing samples from heterogeneous types of B-NHLs (respectively: GSE26673, GSE4475, GSE10172, GSE132929), including the aggressive BLs, have been analyzed. As reported in Table 3, significant positive Pearson and Spearman correlations between *YY1* and *BIRC5* expression levels were found in these GEO datasets (Table 3 and Figure 3). In particular, within a dataset of 16 BLs patients (Piccaluga, GSE26673) it was observed a significantly positive correlation between *YY1* and *BIRC5* expression (Figure 3A). Moreover, a higher, although not significant, *YY1* and *BIRC5* expression was noticeable in children, if compared with adult patients (Figure 3B).

In an expanded dataset (Hummel, GSE4475), 215 mature aggressive B-cell lymphomas patients were stratified into three categories, based on the presence (or absence) of a clear molecular Burkitt’s lymphoma (MBL) signature. The groups were respectively named: MBL (considered proper BL), intermediate-MBL and non-MBL. Interestingly, both *YY1* and *BIRC5* were significantly and positively correlated in all patients (Figure 3C). Strikingly, the MBL patients (green dots) showed a significantly higher expression of both *YY1* and *BIRC5* if compared with the other two groups (intermediate- and negative-MBL, respectively blue and red dots; Figure 3D).

In an additional dataset (Siebert, GSE10172), 36 pediatric mature aggressive B-cell lymphomas patients were divided according to the tumor type into five subgroups. The correlation between *YY1* and *BIRC5* expression was significantly positive in all the subjects (Figure 3E). Importantly, BL and BL-like patients showed a significantly higher expression of both *YY1* and *BIRC5*, when compared to the other patient groups. Once again, the analysis showed that among the mature aggressive B-cell lymphomas, BLs, BLs-like were the tumor subtypes with a significantly higher expression of both *YY1* and *BIRC5* (Figure 3F).

Finally, a dataset of 290 patients affected by different subtypes of B-NHLs (Green, GSE132929) was analyzed. Coherently to the other results, also in this dataset the correlation between *YY1* and *BIRC5* expression was significantly positive when considering all the specimens (Figure 3G). Moreover, when samples were divided into six subgroups according to the tumor type, BL patients showed a significantly higher expression of both *YY1* and *BIRC5*, if compared with less aggressive B-NHLs (including FL, MCL and MZL). Importantly, *BIRC5* was found significantly higher not only in BLs, but also in High-Grade B-NHL (HG-B-NHL), when compared with FL, MCL and MZL (Figure 3H). 

Overall, the bioinformatics results presented in Figure 3 demonstrate that both *YY1* and *BIRC5* are positively correlated within all the B-NHL datasets analyzed, but they appear significantly overexpressed in the more aggressive B-NHLs subsets, and, in particular, within the BL subtype (Table 3 and Figure 3). In conclusion, the levels of expression of both *YY1* and *BIRC5* are relatively higher in BLs subjects, if compared to other B-NHLs which, on the contrary, are considered less aggressive and indolent - including FLs and MCLs.

In order to further assess whether the differences in *YY1* and *BIRC5* expression levels might be used as significant discriminant factors between diverse B-NHL subgroups, Fisher’s exact test and receiver operating characteristics (ROC) analysis have been performed on relevant B-NHL GEO datasets. To conduct the studies, the expression values of both *YY1* and *BIRC5*, in each GEO dataset, have been divided into High-expressing and Low-expressing groups, respectively including samples whose normalized expression was above or below the specific mean value. The contingency tables generated in the studies are summarized in Table S4.

**Figure 4:**
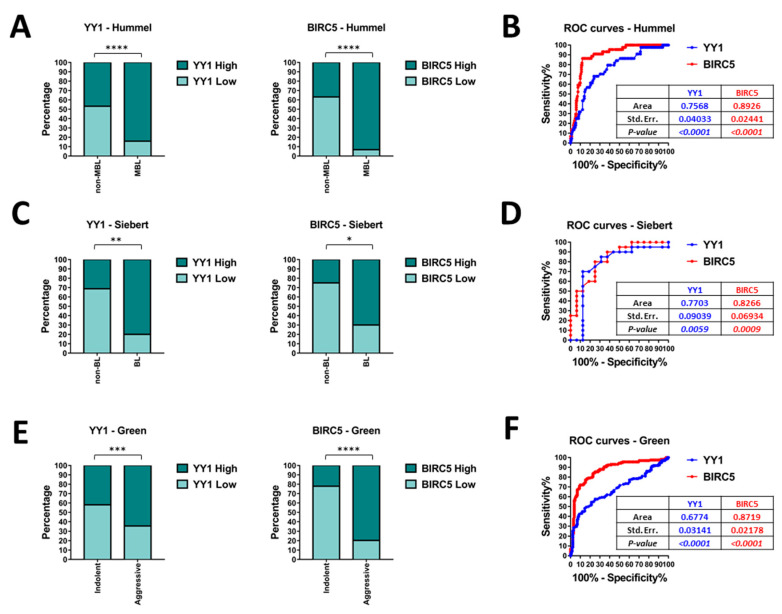
Fisher’s exact test and receiver operating characteristics (ROC) analysis of relevant B-NHL GEO datasets. (**A**). Hummel (GSE4475) GEO dataset Fisher’s exact test for *YY1* and *BIRC5* in MBL and non-MBL subgroups. (**B**). Hummel (GSE4475) GEO dataset ROC analysis of *YY1* (blue) and *BIRC5* (red). (**C**). Siebert (GSE10172) GEO dataset Fisher’s exact test for *YY1* and *BIRC5* in BL and non-BL subgroups. (**D**). Siebert (GSE10172) GEO dataset ROC analysis of *YY1* (blue) and *BIRC5* (red). (**E**). Green (GSE132929) GEO dataset Fisher’s exact test for *YY1* and *BIRC5* in aggressive and indolent B-NHL subgroups. (**F**). Green (GSE132929) GEO dataset ROC analysis of *YY1* (blue) and *BIRC5* (red). The significance in each analysis is presented with asterisks, with * *p* < 0.05; ** *p* < 0.01; *** *p* < 0.001; **** *p* < 0.0001.

Firstly, the Hummel (GSE4475) GEO dataset has been analyzed. Based on the significant differences reported in Figure 3D, the B-NHL samples have been further divided into two subgroups: the MBL (44 samples) and the non-MBL (including the intermediate and the non-MBL samples, 171 in total). As reported in Figure 4A, the contingency analysis through Fisher’s exact test evidenced an extremely significant difference in both *YY1* and *BIRC5* expression, with an 84% of *YY1*-High expressing samples and a 93% *BIRC5*-High expressing samples within the MBL subgroup. Coherently, the ROC curve reported in Figure 4B shows an area under the curve (AUC) of 0.76 for *YY1* and 0.89 for *BIRC5*. These high and significant AUC performances suggest that both *YY1* and *BIRC5* expression levels can be considered as discriminators between MBL and non-MBL diagnostic subgroups.

Moreover, the Siebert (GSE10172) GEO dataset has been analyzed. Based on the results reported in Figure 3D, the samples have been clustered into two main subgroups: the BL subgroup (20 samples, including the BL, BL-like and aggressive-B-NHL) and non-BL subgroup (16 samples, including the DLBCL and FL). As reported in Figure 4C, the contingency analysis through Fisher’s exact test evidenced an extremely significant difference in both *YY1* and *BIRC5* expression, with an 80% of *YY1*-High expressing samples and a 70% *BIRC5*-High expressing samples within the BL subgroup. The ROC curve reported in Figure 4D shows an AUC of 0.77 for *YY1* and 0.83 for *BIRC5*. In agreement with the previous dataset analysis, also in this case, these high and significant AUC performances suggest that both *YY1* and *BIRC5* can be considered as discriminators between BL and non-BL diagnostic subgroups.

Finally, the Green (GSE132929) GEO dataset has been analyzed. The six B-NHL groups were clustered into two separate subgroups, based on the results reported above in Figure 3H. In particular, the aggressive B-NHL subgroup (composed by BLs, HG-B-NHLs and DLBCLs, for a total of 131 samples) and the indolent B-NHL subgroup (including the FL, MCL and MZL subtypes, for a total of 158 samples) have been further analyzed. As reported in Figure 4E, the contingency analysis through Fisher’s exact test evidenced an extremely significant difference in both *YY1* and *BIRC5* expression, with an 65% of *YY1*-High expressing samples and an 80% *BIRC5*-High expressing samples within the aggressive B-NHL subgroup. The ROC curve reported in Figure 4F shows an AUC of 0.68 for *YY1* and 0.87 for *BIRC5*. Analogously to the previous analyses, these high and significant AUC performances suggest that both *YY1* and *BIRC5* can be considered as discriminators between aggressive and non-aggressive diagnostic subgroups.

Overall, the results showed in Figure 4, evidenced that *YY1* and *BIRC5* expression level might be predictive of a specific diagnostic B-NHL subgroup. Notably, *YY1* and *BIRC5* expression levels are significantly higher in the more aggressive subtypes, and, in particular, in BL and BL-like samples. The ROC results suggest a potential diagnostic role for *YY1* and *BIRC5*, as the AUC values in each dataset analyzed were found highly associated with worse B-NHL subtypes, such as BLs.

### 2.4. YY1 Silencing Does Not Affect Raji BL Cellular Growth and It Is Associated with Selective BIRC5 (Survivin) Downregulation

Given the significantly higher expression and positive correlation between *YY1* and *BIRC5*/survivin observed in BLs, a model of aggressive B-NHL, the Raji BL cells have been selected for further validation at cellular and molecular levels. In particular, a shRNA mediated approach has been used to generate three stably transduced Raji-derived cell lines, named respectively KD-01 and KD-02 the two carrying the integrated shRNA against *YY1* transcript, and CTRL the one carrying the shRNA targeting the exogenous luciferase gene, used as mock-non-silencing control. 

**Figure 5:**
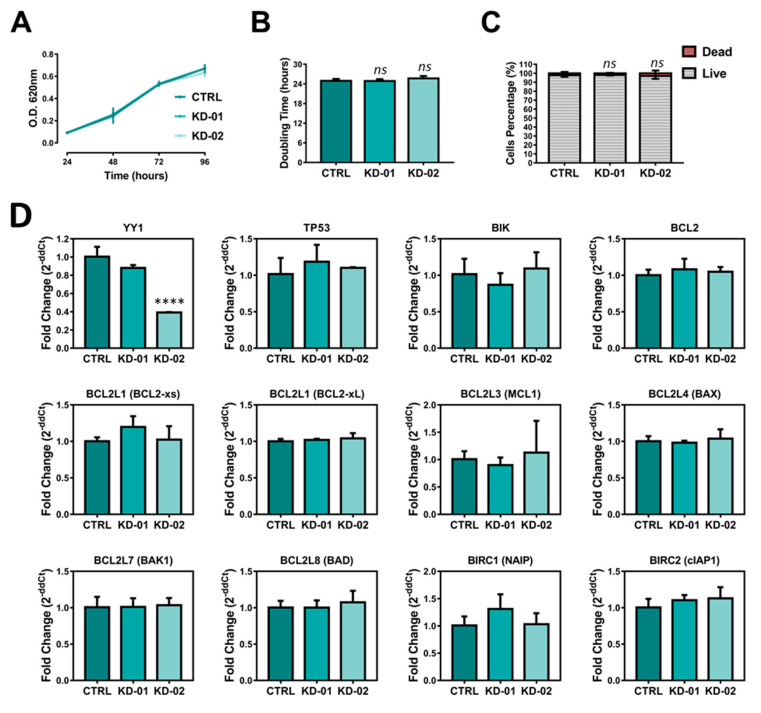

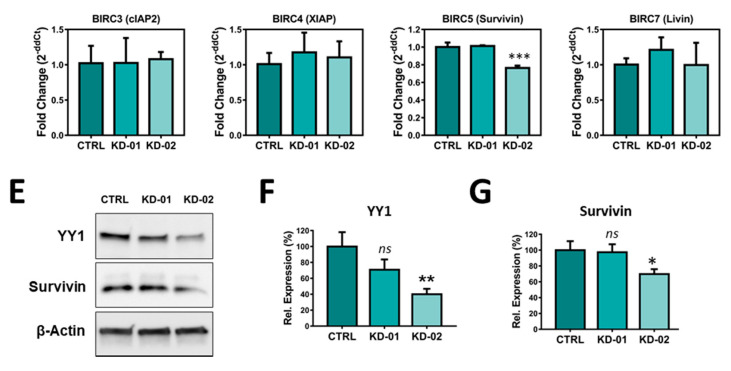
(**A**). MTT-based growth curve of the CTRL, KD-01 and KD-02 Raji cells (OD at 620 nm in function of the days in culture). (**B**). Doubling times of the CTRL, KD-01 and KD-02 Raji cells. (**C**). Viable vs. dead count of Raji CTRL, KD-01 and KD-02, after 96 h in culture (Trypan Blue exclusion method). (**D**). Quantitative-RT-PCR study of *YY1* expression and potential transcriptional *YY1*-target genes in CTRL, KD-01 and KD-02 Raji cells. Each Ct is normalized for the relative Ct of GAPDH (housekeeping control) and then plotted values are expressed as fold change, 2^−ddCt^, compared to CTRL. (**E**). Western blot of CTRL, KD-01 and KD-02 Raji cells for *YY1* (60 KDa), survivin (17 KDa), and β-Actin (42 KDa, normalization control). (**F**). Western blot densitometry analysis for *YY1*. (**G**). Western blot densitometry analysis for survivin. For each sample, the protein signal is normalized to the relative β-Actin signal, and then each value is expressed as percentage compared to CTRL, considered 100%. The results in A, B, C, D, F, G are presented as Mean ± SD; one-way ANOVA with Tukey’s post-hoc test have been performed (* *p* < 0.05; ** *p* < 0.001; *** *p* < 0.001; **** *p* < 0.0001; ns, not significant).

To measure the efficiency of silencing, both the *YY1* transcript and protein expression levels were analyzed in Raji cellular samples. Figure 5D (first upper left plot) shows q-RT-PCR results relative to *YY1* transcript expression, demonstrating that in Raji KD-01 the silencing efficiency was about 12%, with an averaged residual *YY1* mRNA expression of 88% (not significant, *p* = 0.1292), while in Raji KD-02 *YY1* silencing efficiency was about 61%, with an averaged residual *YY1* mRNA expression of 39% (extremely significant, *p* < 0.0001). Furthermore, *YY1* protein levels have been analyzed through immunoblotting. β-Actin was used as normalization control. The immunoblotting images and their densitometric analyses showed a reduction of *YY1* protein expression in both the knocked-down Raji cell lines, when compared with the CTRL, although significant only in the Raji KD-02 (Figure 5E,F).

In order to evaluate whether *YY1* silencing affects Raji cellular growth, MTT and Trypan-Blue growth experiments were performed. The MTT-assay plotted results (the OD was plotted as function of the time in culture) highlighted that the three Raji cell lines showed perfectly overlapping growth curves, without any significant difference (Figure 5A). As shown in Figure 5B, the doubling time for the three cell lines was further calculated, and, accordingly, no significant difference was observed between the KD cells and the CTRL ones. Additionally, a Trypan Blue analysis was performed on the three Raji cell lines. Figure 5C illustrates the live vs. dead percentage analyses, following 96 h in culture. Noticeably, the time in culture did not significantly affect the cellular viability in none of the cell lines tested, with an average of 98% of the population alive vs. a 2% death. Altogether, the observations demonstrate the effectiveness of *YY1* silencing in KD-02 Raji cells, and that *YY1* knock-down does not affect their cellular growth, nor the basal viability.

To validate the positive correlation observed between *YY1* and *BIRC5* in B-NHL patients and, strongly, in BL patients, a RT-PCR-based screening was performed in Raji BL cells. In line with the oncogenic role played by *YY1* in B-NHLs, the downregulation of one (or more) anti-apoptotic factor(s) or the upregulation of one (or more) pro-apoptotic factor(s) is expected in Raji BL cells which are significantly silenced for *YY1* (KD-02). In particular, regarding *BIRC5*, to confirm its positive correlation with *YY1*, a downregulation in Raji BL *YY1*-KD-02 is expected.

In the sq-RT-PCR results reported in Figure S4A, the electrophoretic bands corresponding to the expression, for all the genes selected, in Raji CTRL, KD-01 and KD-02 cells are shown. Among the analyzed genes, both BCL2L2 and BCL2L15 were not expressed in Raji cell lines (the genes are expressed in other cell lines, used as positive controls; data not shown). Strikingly, from the sq-RT-PCR densitometry analysis reported in Figure S4B, it appears that only one gene, *BIRC5*, was selectively downregulated in Raji KD-02 cells compared with Raji CTRL.

Figure 5D illustrates the q-RT-PCR results for the genes which were found to be positively expressed in Raji cells and, for each gene, the relative expression of Raji KD-01 and KD-02 cells was compared with the Raji CTRL expression, considered as unitary (expression of the data are presented as 2^−ddCt^). In agreement with the sq-RT-PCR, from the q-RT-PCR study it emerges that only *BIRC5*/survivin is selectively modulated upon *YY1* silencing. In particular, in Raji KD-02 cells, which are significantly silenced for *YY1*, the expression of *BIRC5* transcript was significantly downregulated of about 24% (residual expression of 76%) compared to CTRL (*p* = 0.0003), whereas in Raji KD-01 cells (which are not significantly silenced for *YY1*) *BIRC5* expression is comparable to CTRL cells. 

In order to assess whether the association between *YY1* silencing and *BIRC5* downregulation is conserved at protein level, an immunoblot analysis has been performed. As shown in Figure 5E,G, in Raji KD-02 cells, survivin protein expression is significantly reduced to 70% compared with the CTRL expression (*p* = 0.0185), while in KD-01 its expression is comparable with the CTRL. In conclusion, as shown in Figure 5, *YY1* silencing in Raji BL cells is associated with survivin downregulation, both at transcriptional and protein levels, therefore suggesting *YY1* as a potential transcriptional regulator of the *BIRC5* gene in BL Raji cells. In turn, such cells recapitulate the positive correlation, as well as the predictive diagnostic value observed for both *YY1* and *BIRC5* in B-NHL patients, and strongly in BLs (Table 3, Figure 3, Figure 4 and Figure S3).

### 2.5. YY1 Silencing Sensitizes Raji BL Cells to Chemotherapy-Induced Cytotoxicity and Enhances Their Apoptotic Response UponTreatments

In order to evaluate whether *YY1* silencing could affect the Raji BL cellular response to drug treatments, and therefore to validate its oncogenic role, Raji CTRL cells and Raji KD-02 cells (significantly silenced for *YY1*) were treated with two drugs, which are part of the first-line protocols used in B-NHL treatment (i.e., CHOP, R-CHOP): doxorubicin and vincristine. Concentration-response curves were setup for each drug, with the MTT assay as viability end-point readout. 

**Figure 6:**
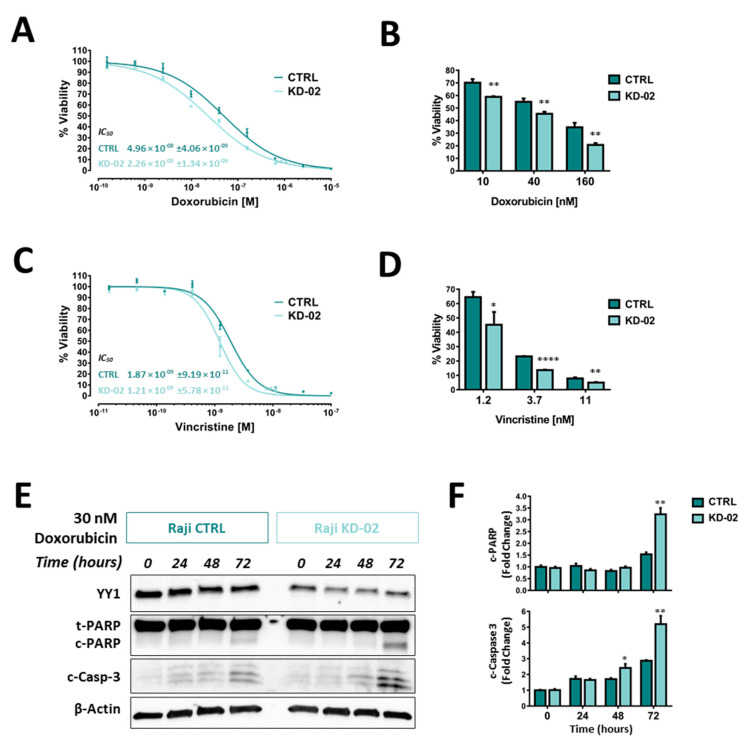

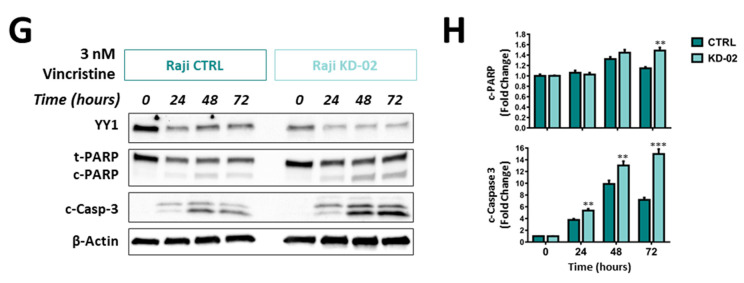
(**A**). Doxorubicin concentration-response treatment in Raji CTRL and KD-02 (knock-down for *YY1*). (**B**). Viability of Raji CTRL and KD-02 at Doxorubicin 10, 40 and 160 nM (doses around the CTRL and KD-02 IC_50_s). (**C**). Vincristine concentration-response treatment in Raji CTRL and KD-02. (**D**). Viability of Raji CTRL and KD-02 at vincristine 1.2, 3.7 and 11 nM (doses around the CTRL and KD-02 IC_50_s). (**E**). Western blot analysis of Raji cells non-silenced (CTRL) and silenced for *YY1* (KD-02), treated with 30 nM doxorubicin, from 0 to 72 h. The signals are detected for *YY1* protein (band at 60 KDa), total PARP (t-PARP) and cleaved PARP (c-PARP) proteins (bands respectively at 89 and 116 KDa), cleaved Caspase 3 (c-Casp-3) protein (two bands at 17 and 19 KDa) and β-Actin protein (used as normalization control, band at 42 KDa). (**F**). Densitometry analysis for c-PARP and c-Casp-3 from the immunoblot reported in A; the data are expressed as fold change of the normalized signals, referred to the 0 h CTRL value. (**G**). Western blot analysis of Raji cells non-silenced (CTRL) and silenced for *YY1* (KD-02), treated with 3 nM vincristine, from 0 to 72 h. The signals are detected for *YY1*, t-PARP and c-PARP, c-Casp-3 and β-Actin proteins. (**H**). Densitometry analysis for c-PARP and c-Casp-3 from the immunoblot reported in C, data expressed as fold change of the normalized signals, referred to the 0 h CTRL value. The results are presented as Mean ± SD, In A and C, the comparison between IC_50_s obtained in CTRL vs. KD-02 cells was performed using the two-tailed unpaired *t*-test (*** *p* < 0.001); in B, D, F, H the comparison between values obtained in CTRL vs. KD-02 cells was performed using the two-tailed unpaired *t*-test with Holm-Sidak correction for multiple comparisons (* *p* < 0.05; ** *p* < 0.01; *** *p* < 0.001; **** *p* < 0.0001).

For the doxorubicin treatment, the curve corresponding to the Raji cells silenced for *YY1* (KD-02) showed a shift towards the left compared with the CTRL, meaning that the Raji cells knocked-down for *YY1* were more sensitive to the doxorubicin treatment. The IC_50_ were respectively 4.96 × 10^−8^ M for Raji CTRL and 2.26 × 10^−8^ M for Raji KD-02 (*p* = 0.0004), being the latter significantly more sensitive to the cytotoxicity induced by doxorubicin (Figure 6A). Figure 6B shows the bars plot for the viability of Raji CTRL and KD-02 at three different concentrations around the IC_50_ (10, 40 and 160 nM) and, for each concentration, the viability was significantly reduced in the KD-02 cells compared with the CTRL (respectively, *p* = 0.0028, *p* = 0.0069, *p* = 0.0035).

Equally, for the vincristine treatment, the curve corresponding to the Raji cells silenced for *YY1* (KD-02) showed a shift towards the left compared with the CTRL, meaning that the Raji cells knocked-down for *YY1* were more sensitive to the vincristine treatment. The IC_50_ were respectively 1.87 × 10^−9^ M for Raji CTRL and 1.21 × 10^−9^ M for Raji KD-02 (*p* = 0.0005), being the latter significantly more sensitive to the cytotoxicity induced by vincristine (Figure 6C). Figure 6D shows the bars plot for the viability of Raji CTRL and KD-02 at three different concentrations around the IC_50_ (1.2; 3.7 and 11 nM) and, for each concentration, the viability is significantly reduced in KD-02 compared with CTRL ones (respectively, *p* = 0.0259, *p* < 0.0001, *p* = 0.0093). 

Altogether, the results shown in Figure 6A–D demonstrate that *YY1* silencing sensitizes Raji B-NHL cells to cytotoxicity induced by two different drug treatments, doxorubicin and vincristine. In fact, for both the drugs tested, when comparing the same concentrations, the KD-02 Raji cells, which are silenced for *YY1*, show a reduction in viability in comparison with the CTRL Raji cells.

Additionally, in order to demonstrate whether the reduction in viability within *YY1* silenced Raji cells was coupled with a concomitant increase in apoptosis induction, a time-course experiment was performed. Figure 6E summarizes the immunoblotting results obtained, following a treatment of 30 nM doxorubicin, which is a concentration close to the calculated IC_50_s (see Figure 6B). Protein samples of Raji CTRL and KD-02 treated cells have been collected at 0, 24, 48 and 72 h post treatment. In order to evaluate the apoptosis activation, the levels of expression of two protein apoptotic markers have been evaluated: cleaved PARP and cleaved Caspase 3. Each marker is detected in apoptotic cells, and their relative levels allow to compare the activation of the apoptotic pathway among different samples. The immunoblotting results suggested that there was a time-dependent increase of both cleaved PARP and cleaved Caspase 3 signals, in both CTRL and KD-02 Raji cell lines. But, by comparing the relative levels of these apoptotic markers within the same time-points within the CTRL vs. KD-02 cells, it is clear that both cleaved PARP and cleaved Caspase 3 signals are higher in KD-02 than in CTRL cells. Therefore, in Raji cells the concentration-dependent decrease in viability is coupled with a time-dependent increase in apoptosis.

The reduction in viability and the concurrent increase in apoptosis are significantly greater in Raji cells which are knocked-down for *YY1* when compared with their unsilenced control. Figure 6F shows the densitometry analysis for cleaved PARP and cleaved Caspase 3. In the bar plot, all normalized signals are expressed as fold change with respect to the normalized CTRL sample at 0 h of treatment, considered as unitary. The densitometry analysis for cleaved PARP at 72 h of treatment shows a significant increase from 1.5 in CTRL to 3.2 folds in KD-02 (*p* = 0.0020). Accordingly, the densitometry analysis for cleaved Caspase 3 shows a significant increase at 48 h of treatment from 1.7 in CTRL to 2.4 folds in KD-02 (*p* = 0.0290), and an increase at 72 h of treatment from 2.9 in CTRL to 5.2 folds in KD-02 (*p* = 0.0067).

Likewise, Figure 6G summarizes the immunoblotting results, following a treatment of 3 nM Vincristine, which is a concentration around the calculated IC_50_s (see Figure 6C). Similarly, cleaved PARP and cleaved Caspase 3 have been evaluated. For vincristine treatments, the immunoblotting results obtained suggest that there is a time-dependent increase of both cleaved PARP and cleaved Caspase 3 signals in both CTRL and KD-02 Raji cell lines. But, by comparing the relative levels of these apoptotic markers within the same time-points, the KD-02 cells show cleaved PARP and cleaved Caspase 3 signals higher in KD-02 than in CTRL cells. Therefore, also for vincristine, the concentration-dependent decrease in viability (reported in the MTT assay, is coupled with a time-dependent increase in apoptosis, which is greater in cells knock-down for *YY1* compared with their unsilenced control. Figure 6H reports the levels of cleaved PARP and cleaved Caspase 3. All normalized signals are expressed as fold change with respect to the normalized CTRL sample at 0 h of treatment, considered as unitary. The densitometry analysis for cleaved PARP at 72 h of treatment shows a significant increase from 1.2 in CTRL to 1.5 folds in KD-02 (*p* = 0.0031). Accordingly, the densitometry analysis for cleaved Caspase 3 shows a significant increase at 24 h of treatment from 3.8 in CTRL to 5.4 folds in KD-02 (*p* = 0.0046), at 48 h of treatment from 9.9 in CTRL to 13.0 folds in KD-02 (*p* = 0.0086), and at 72 h of treatment from 7.2 in CTRL to 15.0 folds in KD-02 (*p* = 0.0005).

In summary, Raji cells which are silenced for *YY1*, when treated with a sub-lethal concentration of either doxorubicin or vincristine, although with different kinetics, showed a time-dependent increase of both cleaved PARP and cleaved Caspase 3 that was significantly greater than the increase detected in CTRL Raji cells, when comparing the same time-points. Altogether, the reported results suggest that *YY1* silencing, in association with a reduction in cellular viability, potentiates the apoptotic response in Raji cells treated with the two different cytotoxic drugs.

### 2.6. YY1 and Survivin Association Is Reverted upon Doxorubicin and Vincristine Treatment Only in BL Raji Cells Which are Efficiently Silenced for YY1

To analyze *YY1* and survivin protein expression variations upon cytotoxic treatments, immunoblotting analyses of both *YY1* and survivin protein levels, following both doxorubicin and vincristine time-course treatments, were performed.

**Figure 7:**
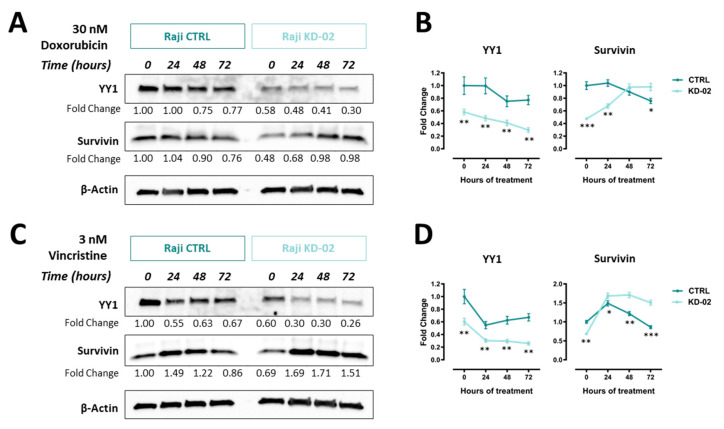
(**A**). Western blot analysis of Raji cells non-silenced (CTRL) and silenced for *YY1* (KD-02), treated with 30 nM doxorubicin, from 0 to 72 h. Signal detected for *YY1* protein (band at 60 KDa), survivin (band at 17 KDa) and β-actin protein (used as normalization control, band at 42KDa). (**B**). Densitometry analysis for *YY1* and survivin from the immunoblot reported in A, data expressed as fold change of the normalized signals, referred to the 0 h CTRL value. (**C**). Western blot analysis of Raji cells non-silenced (CTRL) and silenced for *YY1* (KD-02), treated with 3 nM vincristine, from 0 to 72 h. Signals detected for *YY1*, survivin and β-actin proteins. (**D**). Densitometry analysis for *YY1* and survivin from the immunoblot reported in C, data expressed as fold change of the normalized signals, referred to the 0 h CTRL value. The results reported in B and D are presented as Mean ± SD and the comparison between the values obtained in CTRL vs. KD-02 protein samples was performed using the two-tailed unpaired *t*-test with Holm-Sidak correction for multiple comparisons (* *p* < 0.05; ** *p* < 0.01; *** *p* < 0.001).

Figure 7A shows the results obtained by treating Raji cells with 30 nM doxorubicin, from 0 to 72 h. In particular, for *YY1* it is possible to assess a time-dependent downregulation in terms of total protein, both in CTRL and KD-02 cells. Such levels are significantly lower at 72 h treated samples, when compared with the 0 h samples, both for CTRL and KD-02 cells (Figure S5A). Moreover, this downregulation affects both CTRL and KD-02 Raji cells, therefore, the relative levels of *YY1* were significantly lower in KD-02 vs. CTRL cells, for each time-point analyzed (Figure 7B). 

For survivin, in agreement with the result reported in Figure 5, the protein basal levels are lower in *YY1* knock-down untreated Raji cells (KD-02) when compared to non-silenced control ones (CTRL), thus confirming a positive association between *YY1* and survivin expression at 0 h (Figure 7A). However, upon doxorubicin treatment, for survivin a different kinetic of expression between CTRL and KD-02 cells is observed. In particular, in CTRL cells the survivin levels slightly increased following 24 h of treatment, while at 48 and 72 h decreased, being significantly reduced at 72 h compared with the 0 h time point (Figure S5B). In contrast, in *YY1*-KD-02 Raji cells the survivin levels increased over time, thus being significantly augmented when comparing 0 h vs. 72 h samples (Figure 7B and Figure S5B).

Figure 7C shows the data for 3 nM vincristine treatment, from 0 to 72 h. Likewise, for vincristine, for *YY1* protein it is possible to measure a time-dependent downregulation, both in CTRL and KD-02 cells. *YY1* protein expression is significantly lower at 72 h treated samples, when compared with the 0 h samples, both for CTRL and KD-02 cells (Figure S5C). Moreover, this downregulation affects both CTRL and KD-02 Raji cells; therefore, the relative levels of *YY1* were significantly lower in KD-02 vs. CTRL cells, for each time-point analyzed (Figure 7D). 

Accordingly, for vincristine, in Figure 7C it is possible to observe that the survivin basal levels are lower in *YY1* knock-down untreated Raji cells (KD-02) when compared to non-silenced control ones (CTRL), thus confirming a positive association between *YY1* and survivin expression at 0 h. However, upon vincristine treatment, for survivin a different kinetic of expression within CTRL and KD-02 cells was observed. In particular, in CTRL cells the survivin levels increased following 24 h of treatment, while at 48 and 72 h decreased strongly, being significantly reduced at 72 h compared with the 0 h time point (Figure S5D). In contrast, in *YY1*-KD-02 Raji cells the survivin levels increased over time up to 48 h, being modestly lower at 72 h compared with 48 h of treatment. Importantly, also for vincristine treatment, it is shown a significant increase of survivin protein level when comparing 0 h vs. 72 h samples (Figure 7D and Figure S5D).

Altogether the results reported in Figure 7 and Figure S5 demonstrate that, while there is a positive correlation between *YY1* and survivin protein levels in untreated cells CTRL vs. KD for *YY1*; when the cells undergo apoptosis, such a correlation becomes negative, consistently and selectively in doxorubicin- and vincristine-treated KD-02 Raji cells, following 72 h of treatment. In summary, *YY1* is downregulated in a time-dependent manner in both CTRL and KD-02 Raji cells upon cytotoxic stimuli. However, while survivin is significantly downregulated in CTRL samples, it is significantly upregulated in *YY1*-KD samples, at the latest time point, for both treatments. Therefore, the positive correlation of survivin and *YY1* is selectively reverted only within cells which are knocked-down for *YY1*, thus expressing relative lower levels of *YY1* protein.

## 3. Discussion

*YY1* is a pleiotropic transcription factor, able to modulate more than one pathway within multiple tissues and organs, including the regulation of the apoptosis. *YY1* plays a key role in mouse embryogenesis and tissue development, as its absence in not compatible with life [29]. Moreover, *YY1* haploinsufficiency in humans leads to a severe intellectual disability disorder [30]. Regarding the immune system, *YY1* is known to be essential at all stages of B-cell differentiation [10,31].

Additionally, *YY1* mutation or dysregulation may bring to either its downregulation or overexpression, and the alteration in *YY1* intracellular levels may be linked with a wide range of conditions, including cancer [9,32,33]. The final outcome is strictly context-dependent [9].

Among the tumors in which *YY1* has been seen to be a fundamental factor, are included B-NHLs. When dysregulated, *YY1* may be linked with lymphomagenesis and several reports describe *YY1* as negative prognostic marker in the vast majority of hematological malignancies [12]. Although some studies showed that *YY1* silencing or inhibition might sensitize B-NHL cells to chemotherapy or immunotherapy, the functional role played by such transcription factor at molecular levels still remains elusive [34].

In this work, by using an in-silico promoter regions screening approach (JASPAR) coupled with an in vitro RT-PCR-based validation method, a potential *YY1* target gene, called *BIRC5*, or survivin, has been identified. Survivin belongs to the IAPs family of apoptosis modulators, it is able to promote both apoptosis inhibition and cell cycle progression, and it is highly expressed during G2-M phase of the cell cycle [35]. Although *YY1* has been demonstrated to regulate survivin expression in other models, the mechanism is not totally clarified, as it seems to rely on the specific cancer nature [36].

Firstly, to support the association existing between *YY1* and *BIRC5* gene expression, thus elucidating the potential clinical role played by both factors in B-NHL patients, bioinformatics data obtained from publicly available databases have been analyzed. The analysis of *YY1*-ChIP-Seq experiments deposited on ENCODE corroborates the strong interaction occurring between *YY1* factor and *BIRC5* promoter within different cellular models, including lymphoid cells, thus suggesting a direct binding of *YY1* transcription factor on the survivin promoter.

Importantly, through the use of the R2 platform, *YY1* and *BIRC5* expression levels were further compared in 12 independent B-NHL GEO datasets vs. normal control ones and found significantly higher in tumor specimens. Additionally, the calculated Pearson and Spearman correlations between *YY1* and *BIRC5* expression in all the 12 B-NHL GEO datasets were significantly positive, therefore suggesting a pro-tumorigenic role for both *YY1* and survivin in B-NHL patients. Strikingly, the analyses of mixed B-NHL datasets evidenced that both *YY1* and *BIRC5* were significantly and highly overexpressed in BLs and high-grade B-NHLs, compared to their expression in other milder forms of B-cells lymphomas. Moreover, once the datasets with multiple B-NHL samples were stratified to perform contingency Fisher’s exact tests and ROC analyses, both *YY1* and *BIRC5* were significantly highly expressed in the BL and aggressive B-NHL subgroups. This latter observation clearly suggests that the assessment of *YY1* and *BIRC5*/survivin levels might be used as biomarker of specific aggressive B-NHLs subtypes, especially the widely diffused BLs.

These observations were further validated in Raji cells, which are a widely used BL cellular model. The approach used was a shRNA-mediated silencing strategy, in order to constitutively downregulate the level of *YY1* in Raji cells, which express higher levels of *YY1* compared with normal lymphoid tissues, as previously reported by our group [12]. In this study it was demonstrated that *YY1* silencing does not affect cellular growth and viability. Hence, *YY1* role upon cytotoxic stimuli was further verified.

*YY1* plays an oncogenic role in various hematological tumors. For instance, anti-CD20-mediated *YY1* inhibition sensitizes NHL cells to TRAIL-induced and Fas-induced apoptosis [37,38]. Moreover, *YY1*-RelA complex is able to repress the transcription of the pro-apoptotic factor Bim in MM cells therefore promoting tumorigenesis [39]. Also, *YY1* suppresses miR-let-7a thus driving chemoresistance in AML cells [40]. Coherently, *YY1* is a transcriptional activator of the multidrug resistance gene MDR1, which plays an important role in chemoresistance, in ALL cells [41]. Moreover, it has also been demonstrated that pRb-*YY1* interaction prevents *YY1* binding to the c-Myc promoter in normal B cells, but not in BL cells [13]. Finally, *YY1* may positively regulate the expression of another transcription factor KLF4, showing a dual role in cancer, with a possible prognostic value [42]. Altogether, these findings suggest that the molecular context and the molecular players available (including surrounding co-activators and co-repressors) may highly influence the final outcome of *YY1* transcriptional activity, which can be very heterogeneous.

Importantly, *BIRC5*/survivin has been found to be significantly downregulated, at both transcript and protein levels, in Raji cells which are efficiently silenced for *YY1*. This result validates the positive correlation observed in B-NHL patients. Overall, our findings suggest that survivin might be a potential transcriptional target of *YY1* in Raji BL cells. In light of the JASPAR analysis, as well as the *YY1*-ChIP-seq deposited results, it can be hypothesized that *YY1* might directly promote survivin transcription, also in Raji BL cells. This regulation can be direct, following survivin promoter binding. Alternatively, *YY1* might interact with other transcription factors and co-factors, such as specificity protein 1 (Sp1) transcription factor, and therefore indirectly induce survivin expression [43,44,45].

Interestingly, survivin is an inhibitor of apoptosis, known to regulate mitosis in B cell homeostasis and, importantly, it has a prognostic role in patients with NHL, where its overexpression is coupled with a poorer prognosis [46,47,48]. Correspondingly, it has been demonstrated that the overexpression of survivin initiates hematological malignancies in vivo, while its inhibition suppresses the growth of aggressive forms of NHL [49,50]. Mechanistically, the overexpression of survivin is associated with inhibition of apoptosis initiated via the extrinsic or intrinsic apoptotic pathways. Survivin may interact with effector caspases, thus disrupting the final outcome of the caspase cascade and, in turn, the programmed cell death finalization [51].

Given its overexpression in several cancers coupled with a concomitant lower (or absent) expression in normal tissues, survivin is a widely studied molecular target in oncology, and it has properly considered a golden bullet [52]. The current approaches developed to inhibit survivin in cancer cells can be divided into 5 different classes: (1) survivin-partner protein interaction inhibitors, (2) survivin dimerization inhibitors, (3) survivin gene transcription inhibitors, (4) survivin mRNA inhibitors and (5) survivin immunotherapy. Currently, over 80 studies registered in clinicaltrials.gov are studying both the safety and potential efficacy of complementing traditional anti-cancer approaches with small molecules or survivin-targeting vaccines, thus increasing the overall sensitivity of cancer cells to anti-cancer treatments [52].

The validation experiments performed in Raji BL cells, demonstrate a positive correlation between *YY1* knock-down and survivin downregulation, which is in line with the functional effects observed in terms of viability and apoptosis. In fact, Raji cells silenced for *YY1*, and hence downregulated for survivin, show an increased susceptibility to both doxorubicin and vincristine coupled with an increased overall apoptosis, as shown by the augmented cleavage of the effector Caspase 3 and PARP detected by immunoblotting. Importantly, this result is in further agreement with a previous study from Gu et al., which demonstrated that the direct knock-down of survivin induces apoptosis and growth inhibition in Raji cells [53].

Regarding survivin transcriptional regulation by *YY1*, contradictory results have been reported by others. In fact, it has been shown that *YY1* may inhibit or promote survivin transcription, depending on the molecular milieu of *YY1*-interacting co-activator/co-repressors [36]. While, Galloway et al. [54] found that *YY1* transcriptionally represses survivin gene in an in vitro model of osteosarcoma, Affar et al. [29] showed that survivin levels were decreased in a mouse *YY1*-knock-down model. Consistently with the latter, in a model of colorectal cancer, Zhang and colleagues evidenced that *YY1* expression is positively correlated with survivin expression, as *YY1* knock-down was associated with a downregulation of survivin [55]. The explanation for these opposite findings could lie in the different cellular contexts, which, in general, profoundly change the final effects on the transcriptional modulation of a given gene. An alternative explanation could be the presence of mutated transcription factors’ binding sites along the *BIRC5* promoter in cancer cells, which might affect the overall binding of the various transcriptional regulators, including *YY1* [56].

Our in vitro observations are in agreement with the positive modulation of *YY1* on survivin expression. However, as shown in Figure 7, the time-course experiments performed with both doxorubicin and vincristine, further demonstrated that, following 72 h of cytotoxic stimuli, this positive correlation is significantly inverted, only within cells which are silenced for *YY1* and, hence, expressing relative lower *YY1*, and survivin, basal levels. In particular, we found that *YY1* levels are decreased upon doxorubicin and vincristine treatments, in both CTRL and *YY1*-KD cells. This observation is in line with the results obtained previously by Vega et al., in B-NHL 2F7 cells, where the authors observed a downregulation of *YY1* protein levels following Rituximab (anti-CD20) treatment, and presumably due to NF-kB downregulation [38].

Regarding survivin, while its protein level decreased in Raji CTRL cells following 72 h of either doxorubicin or vincristine treatment, in contrast, within cells effectively knocked-down for *YY1*, survivin protein levels significantly increased upon 72 h of both cytotoxic treatments, thereby reverting the positive association existing between *YY1* and survivin expression in basal conditions. This upregulation of survivin levels following apoptosis, may depend on the long-term effects of the apoptotic response, which have been reported to affect the overall subcellular distribution of survivin. In particular, it has been observed that during apoptosis survivin accumulates in the nucleus, thus being unable to perform its anti-apoptotic function (which is exerted in the cytoplasm). In cells that express less *YY1*, and therefore less survivin, this nuclear accumulation may be relatively more effective, thus justifying the more powerful apoptotic response detected in *YY1*-KD Raji cells (as demonstrated by cleaved Caspase 3 and cleaved PARP immunoblotting results reported in Figure 6) [57,58].

In conclusion, the results reported in this work overall suggest the oncogenic role played by both *YY1* and survivin in aggressive B-NHLs, in particular in BLs, as suggested by the validations studies conducted in Raji cells. These observations might open up to novel research directions, to further explore a potential use of both *YY1* and survivin as markers for diagnosis of aggressive types of B-cells lymphomas. Finally, both *YY1* and survivin may be additionally analyzed in the future as pharmacological targets in the search for new therapeutic approaches for the effective cure of resistant/relapsing B-cells lymphomas.

## 4. Materials and Methods

### 4.1. Determination of YY1 Binding Sites Localized within the Promoters of Apoptosis Regulator Genes by Ensembl Search and JASPAR Analysis

The Ensembl database [59] was used to search for the transcriptional regulatory regions sequences of the main genes involved in apoptosis regulation, including the ones belonging to the B-cell lymphoma 2 (BCL2) family and to the inhibitors of apoptosis proteins (IAPs) family. Subsequently, the analysis for the presence of *YY1* putative binding sites located within the identified transcriptional regulatory regions was performed using JASPAR open-access database, by using the deposited *YY1* binding site matrix profile MA0095.2 [60]. In particular, for each candidate gene, 3000 nucleotides (according to the Ensembl deposited gene sequence) have been analyzed: from 2000 upstream to 1000 downstream the Transcription Starting Site (TSS). As outcome of the analysis, with the selected apoptotic genes, their regulatory elements were analyzed and the promoters’ analysis results are reported in Table S1.

### 4.2. Bioinformatics Analyses

Computational analyses of bioinformatics data contained in three of the biggest bioinformatic portals, the UCSC Genome Browser, ENCODE and Gene Expression Omnibus (GEO) repository, were performed in order to support the in vitro data here obtained [61,62].

First, the “Transcription Factor ChIP-seq” experiments deposited on ENCODE 3, and publicly available on UCSC Genome Browser, were computationally analyzed in order to identify the transcription factors able to bind *BIRC5* promoter and to establish the interaction levels of *YY1* within *BIRC5* promoter in both normal and cancer cell lines [63].

Subsequently the GEO datasets, 12 from B-NHL patients and five from normal heathy subjects, reported in Table 2, were analyzed by using the R2 Genomics Analysis and Visualization Platform to establish the expression levels of *YY1* and *BIRC5* in B-NHL patients compared to normal controls and to study the correlation between *YY1* and *BIRC5* in B-NHL patients datasets [64].

### 4.3. Cell Lines and Culture

The Raji cell line was purchased from the American Type Culture Collection (ATCC, Manassas, VA, USA) and the cells were grown in RPMI 1640 (Sigma-Aldrich, St. Louis, MO, USA). The 293-LinX-A packaging cell line (kind gift from Dr Roberta Maestro, Aviano, Italy) was cultured in DMEM (Sigma-Aldrich, St. Louis, MO, USA). All culture media were supplemented with 2 mmol/lLL-glutamine, 100 IU penicillin, 100 μg/mL streptomycin and 10% heat-inactivated Fetal Bovine Serum (Sigma-Aldrich, St. Louis, MO, USA). All cells were maintained in a constantly humidified, 37 °C and 5% CO_2_ incubator. Mycoplasma absence was assessed by a PCR Assay. All cells investigated in this study were used within 15 passages after thawing.

### 4.4. Generation of Raji Cells Constitutively Silenced for YY1

In order to characterize the functional role played by the *YY1* transcription factor in Raji cells, a shRNA-mediated approach has been used. Three retroviral plasmid vectors have been used. The specific 21 bases-long targeting sequences are: 5′-GCTCTGTAATCTCGTTTCAAA-3′ for KD-01, directed against the 3′ untranslated region (UTR) of *YY1* mRNA, and 5′-CCTCCTGATTATTCAGA ATAT-3′ for KD-02, directed against the coding sequence (CDS) of *YY1* mRNA. Additionally, a retroviral plasmid vector, carrying a non-targeting shRNA sequence, 5′-CCGCCTGAAGTCT CTGATTAA-3′, directed against an unrelated mRNA from firefly luciferase, has been used as non-targeting control (CTRL). In particular, the three selected shRNA sequences, within the plasmid vector, are inserted inside the 3′ and 5′ flanking regions of human miR30 microRNA, to form a shRNA-mir, under the transcriptional control of the constitutive MSCV-LTR promoter (Figure S6).

*YY1* silencing retroviral vectors, pSMP-*YY1*_1 and pSMP-*YY1*_2 and non-silencing control pSMP-Luc were generated by George Daley and deposited in the Addgene plasmid bank (respectively addgene-36357, addgene-36358; addgene-36394, Addgene, Watertown, MA, USA) [65]. pSMP-*YY1*_1, pSMP-*YY1*_2 and pSMP-Luc retroviral particles were prepared using the amphotropic packaging cell line 293-LinX-A, according with the published protocol [66,67,68]. Retroviral supernatants were collected 48 h post-transfection, filtered and stored at −80 °C, until further usage. Retroviral transduction was performed on Raji target cells, following the published protocol [66,67,68]. Transduced cells were selected with Puromycin (1 µg/mL final, Sigma-Aldrich, St. Louis, MO, USA) added to the culture medium until positive selection was accomplished (7 days). Puromycin-selected cellular populations were harvested for expansion and further analyses were performed (total RNA extraction, total protein extraction, viability assays and dose-response assays). Three stably pooled transduced cell lines were established, one per each construct, and called respectively: Raji KD-01 and Raji KD-02 the two cell lines carrying the shRNA targeting *YY1* transcript, and Raji CTRL, the cell line carrying the non-targeting shRNA. Each resulting cellular pool was composed of cells with a random and stable genomic integration of the retroviral construct.

### 4.5. Cell Viability Assay

The 3-(4,5-dimethylthiazol-2-yl)-2,5-diphenyl tetrazolium bromide (MTT, Sigma-Aldrich, St. Louis, MO, USA) viability assay was used to assess cellular viability for Raji parental, Raji *YY1*-silenced and Raji non-silencing control cells. For the growth curve, cells were seeded in triplicate samples into a 96-well plate at different seeding densities (from 1000 to 8000 cells per well), plates were stamped in 5 replicates, and cellular viability was assessed every day for 5 days. For anti-cancer treatments, cells were seeded in triplicate samples into a 96-well plate at 5000 cells per well. 24 h after seeding, cells were treated with doxorubicin and vincristine, two components of the first-line used protocols for the treatment of NHL were used (Sigma-Aldrich, St. Louis, MO, USA). Dose-response curves were setup for each drug, with MTT assay as readout. Doxorubicin from 1.00 × 10^−5^ to 1.53 × 10^−10^ M (9 serial dilutions 1:4) and vincristine from 1.00 × 10^−7^ to 1.52 × 10^−11^ M (9 serial dilutions 1:3). 72 h after the treatments, cells were assessed for their viability. Viability assessment was done by adding MTT 0.5 µg/mL final to each well. Cells were incubated for 4 h at 37 °C. The insoluble formazan crystals were dissolved by adding acid-isopropanol stop solution (Isopropanol with 0.04 N HCl final) and then by pipetting up and down vigorously. The absorbance was measured at 620 nm, using a Sunrise microplate reader (Tecan, Männedorf, Switzerland). Cell viability was expressed as a percentage compared to control cells, assumed to be 100% viable.

### 4.6. Trypan Blue Cell Count

Cellular samples were mixed 1:1 with 0.4% Trypan Blue (Thermo Fisher Scientific, Waltham, MA, USA) by gently pipetting, and then 20 μL of the mix were loaded into a chamber of the hemocytometer (Bürker chamber). Counts were performed in triplicate using the Eclipse Ts2 inverted microscope (Nikon, Melville, NY, USA). Cells permeable to Trypan Blue were counted as death. Cells perfectly rounded and not permeable to Trypan Blue were considered viable.

### 4.7. Total RNA Extraction, cDNA Synthesis and Semiquantitative and Quantitative RT-PCR Analysis

For total RNA extraction, up to 3 million of cells were harvested and total RNA was isolated using GeneJET RNA Purification Kit (Thermo Fisher Scientific, Waltham, MA, USA), according to manufacturer’s instructions. For cDNA synthesis, 3 µg of the total RNA was reverse-transcribed with Super-Script IV Reverse Transcriptase (Thermo Fisher Scientific, Waltham, MA, USA), using 50 μM random hexamers (Thermo Fisher Scientific, Waltham, MA, USA).

For RT-qPCR the Luminaris Color HiGreen qPCR Master Mix, high ROX (Thermo Fisher Scientific, Waltham, MA, USA) was used. Amplified cDNA levels were quantitatively determined with a 7300 Real-Time PCR System (Applied Biosystems, part of Thermo Fisher Scientific, Waltham, MA, USA). The template cDNA was amplified using the primer pairs reported in Table 4, all designed by using Primer-Blast priming designing tool from NCBI [69].

The real-time PCR program for quantitative RT-PCR (q-RT-PCR) was the following: UDG pre-treatment at 50 °C for 2 min, followed by an initial denaturation step at 95 °C for 10 min and a 3-step PCR program at 95 °C for 15 s, 60 °C for 30 s and 72 °C for 30 s, for 40 cycles. The expression levels of target genes were normalized to the averaged expression levels of human GAPDH, used as the housekeeping gene.

For semi-quantitative RT-PCR (sq-RT-PCR), DreamTaq Green PCR Master Mix, Thermo Fisher Scientific, Waltham, MA, USA) was used. The template cDNA was amplified using the primer pairs reported in Table 4 (same as in q-RT-PCR). The PCR program was: initial denaturation step at 95 °C for 2 min and a 3-step PCR program at 95 °C for 30 s, 60 °C for 30 s and 72 °C for 1 min, final elongation at 72 °C for 5 min. PCR products were analyzed through gel electrophoresis and the expression levels of target genes in each sample was normalized to the expression levels of human GAPDH in the same sample, used as the housekeeping gene.

### 4.8. Protein Lysates Preparation, Quantification and Immunoblotting Analysis

For protein extraction, up to 5 million of cells were harvested. The collected cells were lysed using nonidet-P40 (NP40) buffer (150 mM NaCl, 1.0% NP-40, pH 8.0 50 mM Tris, Thermo Fisher Scientific, Waltham, MA, USA) supplemented with protease inhibitors and phosphatase inhibitors (Roche Diagnostics, Indianapolis, IN, USA). Protein concentration was determined using the Bradford assay (Bio-Rad Laboratories, Hercules, CA, USA), according to manufacturer’s instructions. Protein samples (30 µg of total protein extract per sample) were separated using Mini-PROTEAN TGX Precast Gels and Mini-PROTEAN gel-electrophoresis system (Bio-Rad Laboratories, Hercules, CA, USA). Protein gels were then transferred onto a nitrocellulose membrane using the TransBlot Turbo transfer system (Bio-Rad Laboratories, Hercules, CA, USA). The membranes were blocked for 1 h at room temperature (RT) with 5% of non-fat dry milk diluted in TBS-T buffer (0.1% Tween 20, 20 mM Tris–HCl pH 7.6, 137 mM NaCl). Immunoblotting analysis was performed using the following antibodies, according to manufacturer’s instructions: anti-*YY1* (Rabbit, Cell Signaling Technology, Danvers, MA, USA, CST-2185); anti-β-Actin (Mouse, Sigma-Aldrich, St. Louis, MO, USA, a-1978); anti-PARP (Rabbit, Cell Signaling Technology, Danvers, MA, USA, CST-9532); anti-cleaved-Caspase-3 (Rabbit, Cell Signaling Technology, Danvers, MA, USA, CST-9664), anti-GAPDH (Mouse, Santa Cruz Biotech, Dallas, TX, USA, sc-137179), anti-H3-Histone (Rabbit, Abcam, Cambridge, UK, ab1791), anti-Survivin (Rabbit, Cell Signaling Technology, Danvers, MA, USA, CST-2808); Goat Anti-Rabbit IgG Antibody, Fc, HRP conjugate (Chemicon International, Fisher Scientific, Waltham, MA, USA, AP156P), Goat Anti-Mouse IgG Antibody, Fc, HRP conjugate (Chemicon International, Fisher Scientific, Waltham, MA, USA, AP127P). Membrane signal was detected incubating the stained membrane with the enhanced chemiluminescence (ECL) kit (Bio-Rad Laboratories, Hercules, CA, USA), following manufacturer’s instructions. ECL developed membranes were acquired using the ChemiDoc Touch Imaging System (Bio-Rad Laboratories, Hercules, CA, USA).

### 4.9. Statistical Analyses

The experiments were performed in triplicates. Statistical analyses were performed using GraphPad Prism version 7.0 for Windows (GraphPad Software, La Jolla, CA, USA). The results were presented as average ± standard deviation (SD) or as median ± SD, as indicated in each figure accordingly. Comparisons between three groups has been performed using one-way analysis of variance (ANOVA) with Tukey’s post-hoc test. Multiple comparisons between two groups were performed using the two-tailed unpaired *t*-test with Holm-Sidak post-hoc test. Comparisons between two groups on a single parameter were conducted using the two-tailed unpaired *t*-test.

The normalized expression value distribution of both *YY1* and *BIRC5* (respectively from *YY1* 200047_s_at and *BIRC5* 202095_s_at probes normalized reads) in the 12 Tumor GEO Datasets has been evaluated with a D’Agostino and Pearson normality test.

The contingency analysis of the relevant GEO datasets including several different B-NHL subgroups has been performed by using the Fisher’s exact test (odds ratio calculation with Baptista-Pike posttest). While the ROC curve analysis and AUC calculation has been used to further predict the *YY1* and *BIRC5* diagnostic relevance in such datasets, comparing per each obtained contingency table the *YY1* or *BIRC5* gene expression within the two diagnostic subgroups. The statistical significance in figures and tables has been indicated by asterisks, being * = *p* < 0.05; ** = *p* < 0.01; *** = *p* < 0.001; **** = *p* < 0.0001, while n.s. stands for not significant where *p* > 0.05.

## Figures and Tables

**Table 1:** JASPAR analysis results for *YY1* binding sites located within the promoter of *BIRC5* gene (Sequence ID: GRCh38:17:78212186:78226236:1).

Relative Score	Start	End	Strand	Predicted Sequence
0.845	770	781	+	AAACATGGTGAA
0.845	155	166	−	CACCATGGCCTC
0.807	2491	2502	+	GCAGATGGCCGA
0.804	2819	2830	+	TAAGATGCCTGA
0.804	1002	1013	+	AAGAATGGGGGC

**Table 2:** Features of the GEO Datasets (Data normalization used: MAS5.0).

GEO ID	Method	Samples Type	Samples	Contributor	Reference
GSE26673	u133p2	T-BL	16	Piccaluga PP	[15]
GSE132929	u133p2	T-B-NHL (BL-59; DLBCL-95; FL-65; HGBCL-4; MCL-43; MZL-23)	290	Green MR	na
GSE10846	u133p2	T-DLBCL	420	Xiao W	[16]
GSE56315	u133p2	T-DLBCL	122	Boedker JS	[17]
GSE87371	u133p2	T-DLBCL	223	Jardin F	[18]
GSE81183	u133p2	T-FL	42	McKeithan TW	[19]
GSE93261	u133p2	T-FL	149	Salles G	[20]
GSE16024	u133p2	T-B-NHL (7-MCL; 7-FL)	14	Du MQ	na
GSE93291	u133p2	T-MCL	122	Staudt LM	[21]
GSE7307	u133p2	N-Various tissues	504	Roth RB	na
GSE3526	u133p2	N-Various tissues	353	Roth RB	[22]
GSE46510	u133p2	N-PBC	154	Lye SJ	[23]
GSE4475	u133a	T-BL	215	Hummel M	[24]
GSE10172	u133a	T-Pediatric ma-B-NHL (8-BL; 5- BL like; 14-DLBCL; 2-FL; 7-AU B-NHL)	36	Siebert R	[25]
GSE57611	u133a	T-DLBCL	148	Kreuz M	[26]
GSE1133	u133a	N-Various tissues	158	Su AI	[27]
GSE3846	u133a	N-PBC	108	Baty F	[28]

**Abbreviations:** u133p2, Affymetrix HU133 plus 2.0 microarray; u133a, Affymetrix HU133a microarray; T, Tumor; N, Normal; BL, Burkitt’s Lymphoma; B-NHL, B-cells non-Hodgkin’s Lymphoma; DLBCL, Diffused Large B-cells Lymphoma; FL, Follicular Lymphoma; HGBCL, High-grade B-cell Lymphoma; MCL, Mantle Cells Lymphoma; MZL, Marginal Zone Lymphoma; PBC, Peripheral Blood Cells; maB-NHL, Mature Aggressive B-NHL; AU, Aggressive Unclassified; na, not associated.

**Table 3:** *YY1* and *BIRC5* Normality test, Pearson and Spearman correlation (R-Value) results.

GEO ID	Analyzed Samples	*YY1* Normality Test	*BIRC5* Normality Test	Pearson R	Pearson P	Spearman R	Spearman P
GSE93261	149	0.3874 (Y)	0.0013 (N)	0.2658	0.0011 (**)	0.2523	0.0019 (**)
GSE81183	42	0.0001 (N)	0.4082 (Y)	0.4293	0.0046 (**)	0.3948	0.0097 (**)
GSE16024	14	0.0049 (N)	0.0042 (N)	0.7882	0.0008 (***)	0.5413	0.0481 (*)
GSE93291	122	0.0843 (Y)	0.0001 (N)	0.3638	<0.0001 (****)	0.3926	<0.0001 (****)
GSE87371	223	0.3301 (Y)	0.2087 (Y)	0.572	<0.0001 (****)	0.5937	<0.0001 (****)
GSE57611	116	0.2843 (Y)	0.8101 (Y)	0.2379	0.0101 (*)	0.226	0.0147 (*)
GSE56315	89	0.2221 (Y)	0.3798 (Y)	0.4523	<0.0001 (****)	0.4365	<0.0001 (****)
GSE10846	414	0.7196 (Y)	0.0414 (N)	0.5321	<0.0001 (****)	0.5524	<0.0001 (****)
GSE26673	16	0.4792 (Y)	0.4778 (Y)	0.7747	0.0004 (***)	0.7491	0.0012 (**)
GSE4475	215	0.0001 (N)	0.3127 (Y)	0.2204	0.0011 (**)	0.2781	<0.0001 (****)
GSE10172	36	0.3155 (Y)	0.9022 (Y)	0.498	0.002 (**)	0.6044	<0.0001 (****)
GSE132929	290	0.0001 (N)	0.0001 (N)	0.6306	<0.0001 (****)	0.5355	<0.0001 (****)

**Abbreviations:** Y, Yes; N, No. * *p* < 0.05; ** *p* < 0.01; *** *p* < 0.001; **** *p* < 0.0001.

**Table 4:** Primers used for RT-PCR analyses of genes regulators within the apoptotic pathway.

GENE ID	Primer F	Primer R	bp
*BCL2*	TGAACTGGGGGAGGATTGTG	CGTACAGTTCCACAAAGGCA	183
*BCL2L1 02*	AGCTTTGAACAGGATACTTTTGTGG	GGTGGGAGGGTAGAGTGGAT	183
*BCL2L1 01*	CTGTGCGTGGAAAGCGTAGA	GCTGCTGCATTGTTCCCATAG	155
*BCL2L2*	CACCCAGGTCTCCGATGAAC	GCTGTGAACTCCGCCCAG	210
*BCL2L3*	TTTTCAGCGACGGCGTAACA	CAAACCCATCCCAGCCTCTTT	189
*BCL2L4*	CCCCGAGAGGTCTTTTTCCG	TGGTTCTGATCAGTTCCGGC	145
*BCL2L7*	GATCCCGGCAGGCTGATCC	GTAGCTGCGGAAAACCTCCT	156
*BCL2L8*	CTTTAAGAAGGGACTTCCTCGC	GTGGAGTTTCGGGATGTGGA	163
*BCL2L15*	ACCTGGTGTGCTCAGGATTC	TCCAGATTTTCCCAACCTCCC	194
*BIRC1*	TCAAGCCGTCCCATTTGTTG	TGCTGACACTGCTGGATGAT	204
*BIRC2*	AAGTGGTTTCCAAGGTGTGAGT	AAGCCCATTTCCAAGGCAGATT	230
*BIRC3*	TCTGGGCAGCAGGTTTACAA	GCATTCTTTGGATAGTAAAACACCA	191
*BIRC4*	TGTCCTGGCGCGAAAAGGT	CGTGCCAGTGTTGATGCTGA	190
*BIRC5*	CAAGGACCACCGCATCTCTA	TGTTCCTCTATGGGGTCGTCA	189
*BIRC7*	GGCTCTGAGGAGTTGCGTCT	CTGATGGCCTGTGTGGAAGAAG	105
*BIK*	CCGCCAGAGGAGAAATGTCTGA	TCCTCCATAGGGTCCAGGTC	145
*TP53*	CCCCTCCTCAGCATCTTATCC	GTACAGTCAGAGCCAACCTCAG	124
*YY1*	GAGAGAACTCACCTCCTGAT	GGCTTCTCTCCAGTATGAAC	325
*GAPDH*	AGAAGGCTGGGGCTCATTTG	AGGGGCCATCCACAGTCTTC	258

## Supplementary Materials

Supplementary materials can be found at https://www.mdpi.com/1422-0067/21/17/6446/s1.

## Abbreviations

B-NHLs	B-cell non-Hodgkin lymphomas
*YY1*	Yin Yang 1
GEO	Gene Expression Omnibus
BLs	Burkitt’s lymphomas
WHO	World Health Organization
DLBCL	Diffuse large B-cell lymphoma
FL	Follicular lymphoma
MCL	Mantle Cells lymphoma
MZL	Marginal Zone lymphoma
PcG	Polycomb Group
TSS	Transcription Starting Site
