# Supplementary material for: Role of the Transcription Factor Yin Yang 1 and Its Selectively Identified Target Survivin in High-Grade B-Cells Non-Hodgkin Lymphomas: Potential Diagnostic and Therapeutic Targets
Source: Int J Mol Sci. 2020 Sep 3;21(17):6446. doi: 10.3390/ijms21176446 (PMC7504013; doi:10.3390/ijms21176446)
Supplement: Supplementary file 1 [file ijms-21-06446-s001.pdf]

Figure S1. *Whole Transcription Factors ChIP-seq binding-clusters within BIRC5 promoter, according to ENCODE.* Gray boxes identify each peak cluster relative to transcription factors occupancy within BIRC5 promoter. The length of each box indicates the region of binding within the promoter, while the gray-scale of the boxes is proportional to the strength of the binding to the promoter per each identified transcription factor. Near each box cluster are reported: (1) the ratio between the number of cell lines where the transcription factor positively binds the promoter over the total number of cell lines tested; (2) the ENCODE cell type abbreviation of cells where the positive binding has been reported.

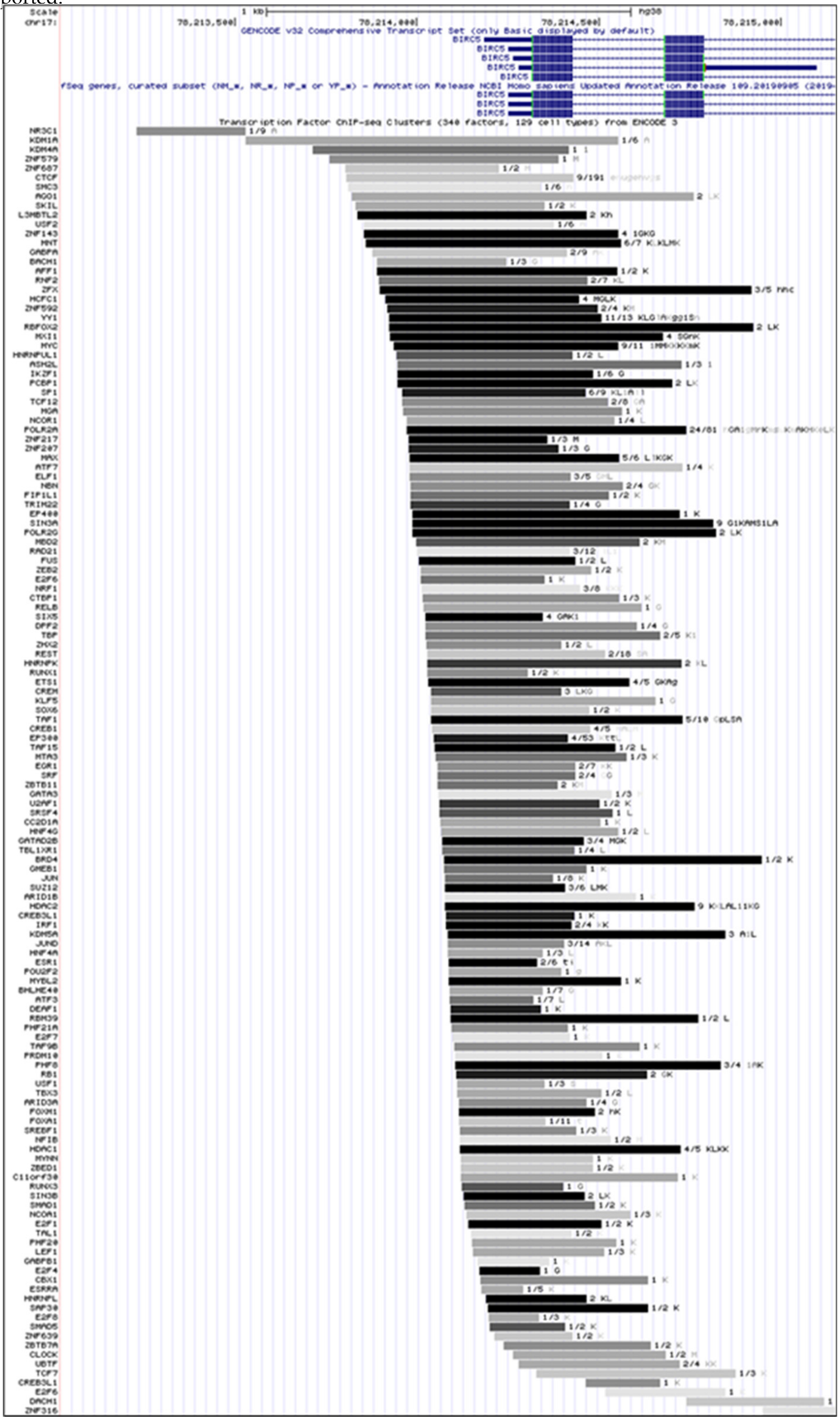

Figure S2. *YY1 and BIRC5 expression values distribution.* **A.** Normalized expression box plots showing YY1 and BIRC5 expression in the 12 B-NHL GEO datasets. **B.** Histogram distribution of YY1 and BIRC5 expression values in the 12 B-NHL GEO datasets. YY1 200047_s_at (blue) and BIRC5 202095_s_at (red) were the probes used.

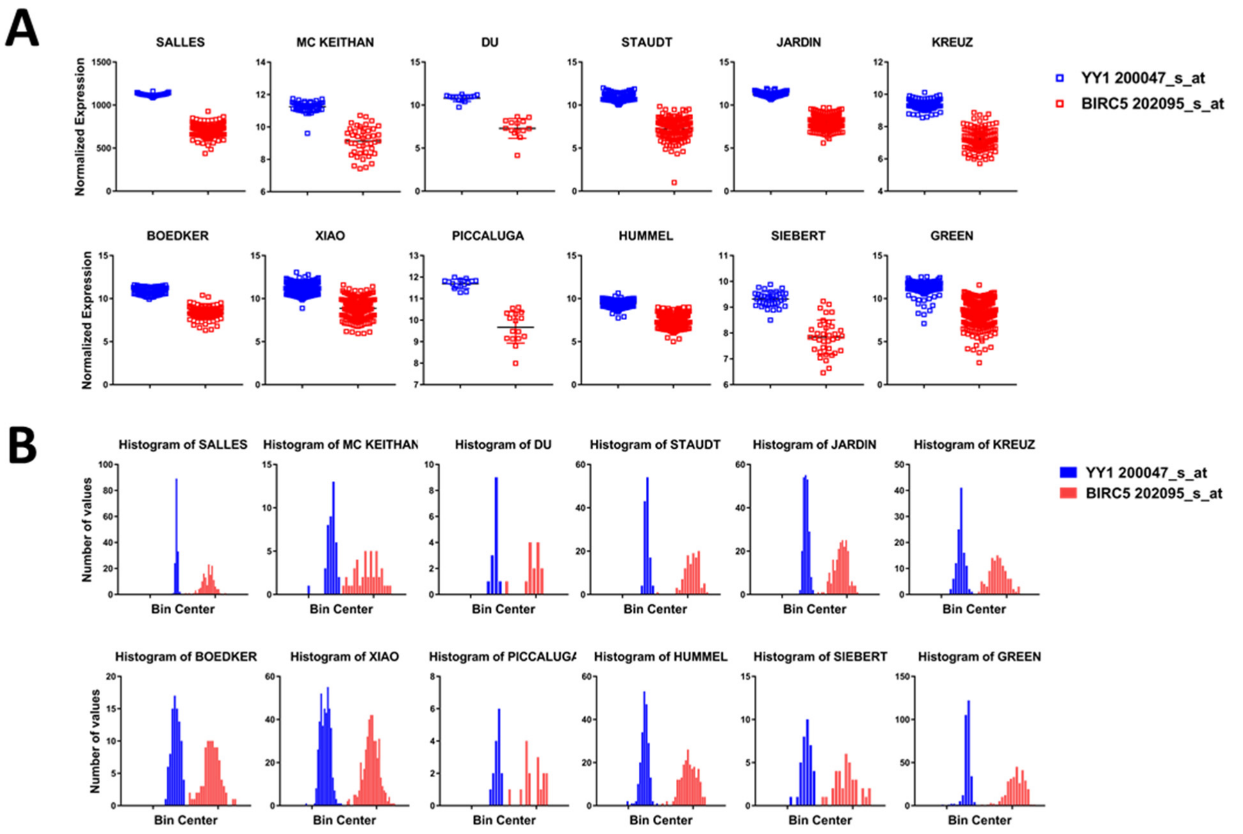

Figure S3. *Correlation between YY1 and BIRC5 expression from the R2 analysis of 8 different GEO B-NHL datasets and relative YY1 and BIRC5 expression when samples are subdivided in Stages and Grades subgroups.* **A.** GSE93261, FL, Follicular Lymphoma. **B.** GSE81183, FL. **C.** GSE16024, FL and MCL, Mantle Cells Lymphoma. **D.** GSE93291, MCL. **E.** GSE87371, Diffused Large B-cells Lymphoma, DLBCL, patients stratified according to the Stages (I, red; II, yellow, III, blue, IV, grey). **F.** GSE87371. Relative expression of YY1 in the 4 Stages. **G.** GSE87371. Relative expression of BIRC5 in the 4 Stages. **H.** GSE57611. DLBCL, patients stratified according to GCB/ABC diagnosis (orange, ABC; blue, GCB; grey, unclassified). **I.** GSE57611. Relative expression of YY1 in the different diagnosis subgroups. **J.** GSE57611. Relative expression of BIRC5 in the different diagnosis subgroups. **K.** GSE56315, DLBCL, patients stratified according to GCB/ABC diagnosis (orange, ABC; blue, GCB; grey, unclassified). **L.** GSE56315. Relative expression of YY1 in the different diagnosis subgroups. **M.** GSE56315. Relative expression of BIRC5 in the different diagnosis subgroups. **N.** GSE10846, DLBCL, patients stratified according to GCB/ABC diagnosis (orange, ABC; blue, GCB; grey, unclassified). **O.** GSE10846, Relative expression of YY1 in the different diagnosis subgroups. **P.** GSE10846. Relative expression of BIRC5 in the different diagnosis subgroups. In F, G, I, J, L, M, O, P data are presented as dotted plots with Median ± SD. Significance was evaluated using one-way ANOVA with Tukey's post-hoc comparison test (the differences are not significant, *ns*).

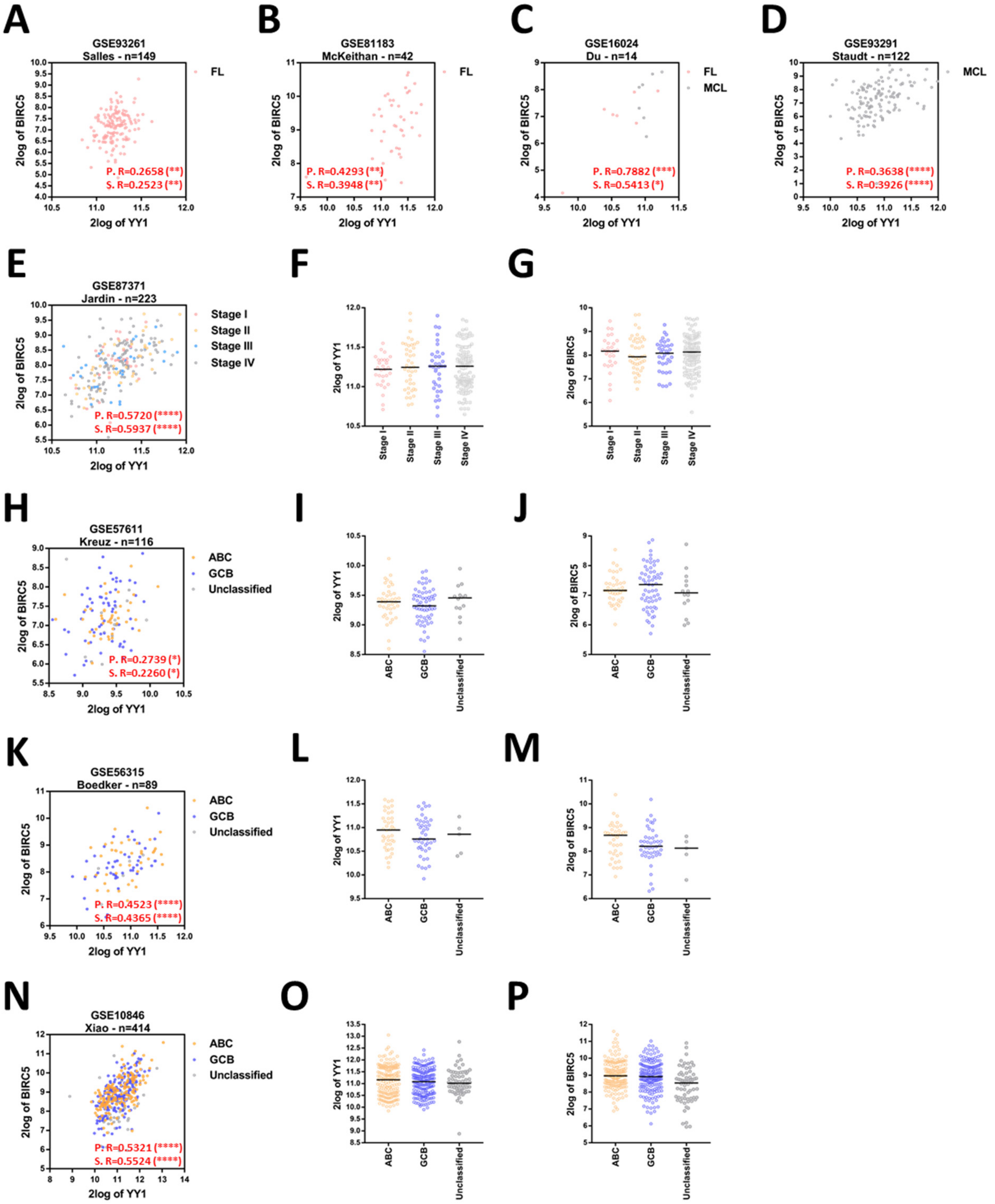

Figure S4. *sq-RT-PCR analysis of YY1 putative transcriptional targets in the apoptotic pathway in Raji CTRL, Raji KD-01 and Raji KD-02 cells.* **A**. Gel electrophoresis results. **B.** Densitometry analysis of sq-RT-PCR bands, each signal is normalized with GAPDH, used as housekeeping. The results are presented as Mean ± SD and one-way ANOVA with Dunnett's post-hoc test have been performed (** P < 0.01; where otherwise reported the test is not significant).

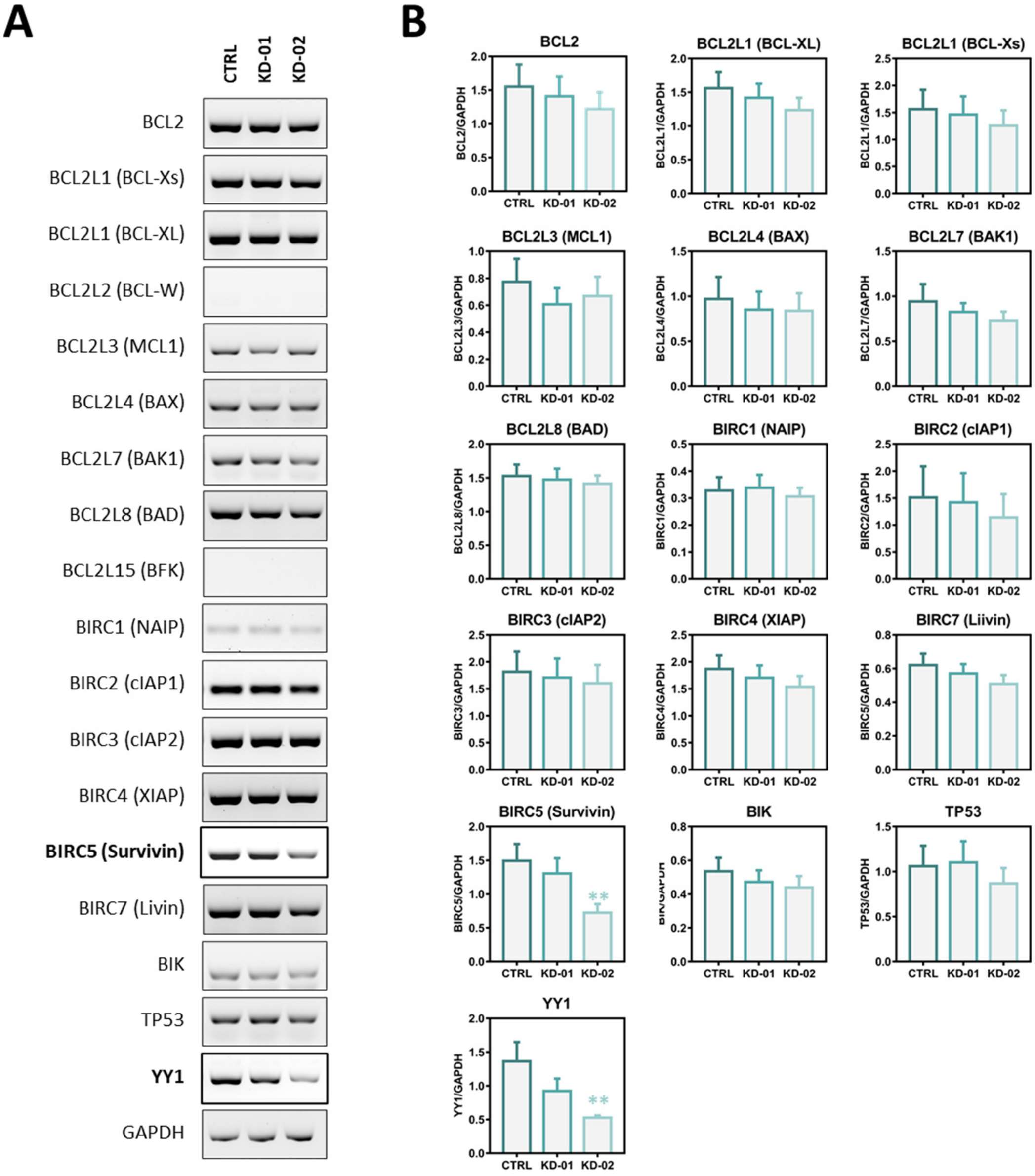

Figure S5. *Densitometry analysis for YY1 and survivin from the immunoblotting reported in Figure 6.* **A.** YY1 protein levels in 0 *vs.* 72 hours Doxorubicin treated samples. **B.** Survivin protein levels in 0 *vs.* 72 hours Doxorubicin treated samples. **C.** YY1 protein levels in 0 *vs.* 72 hours Vincristine treated samples. **D.** Survivin protein levels in 0 *vs.* 72 hours Vincristine treated samples. Data are expressed as fold change of the normalized signals, referred to the 0 hours CTRL value. The results are presented as Mean ± SD and the comparison between the values obtained in CTRL *vs.* KD-02 protein samples was performed using the two-tailed unpaired t-test with Holm-Sidak correction for multiple comparisons (*P < 0.05; ** P < 0.01; *** P < 0.001; **** P<0.0001).

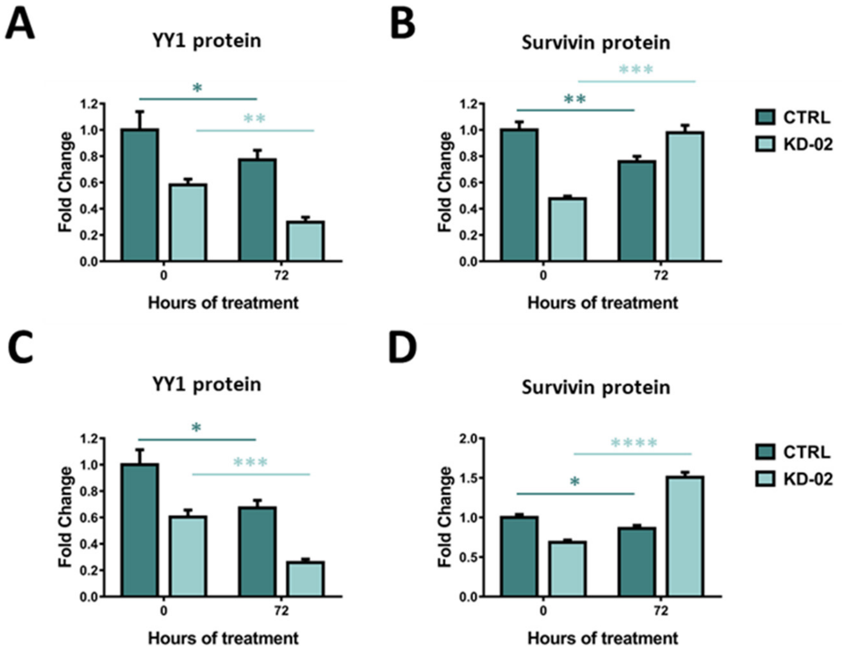

Figure S6. *Retroviral pSMP plasmid features.* With miR-30 hairpin, which includes the specific 21-mers against selected targets, reported in lower part of the figure, with specific hairpin sequences for pSMP-YY1_1, pSMP-YY1_2 and pSMP-LUC.

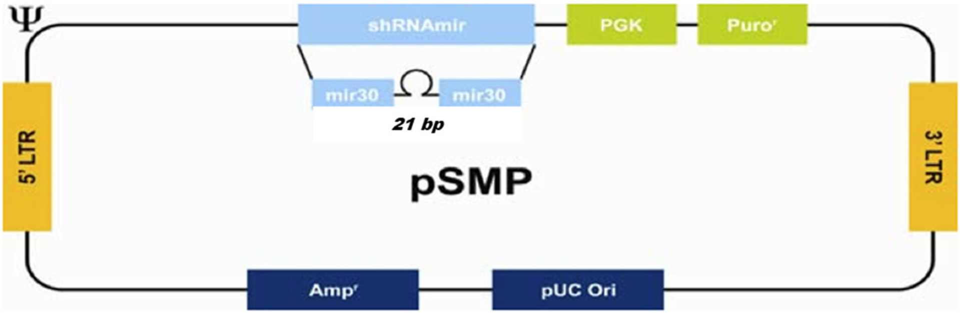

shRNA TARGETING SEQUENCES (21bp):

KD-01 GCTCTGTAATCTCGTTTCAAA

KD-02 CCTCCTGATTATTCAGAATAT

CTRL CCGCCTGAAGTCTCTGATTAA

shRNAmir sequences:

KD-01: PSMP-YY1_1 (NM_003403.5; EXON 5 – 1164-6534, 3'-UTR, 1552-1570)

TGCTGTTGACAGTGAGCGCGCTCTGTAATCTCGTTTCAAA**TAGTGAAGCCACAGATGTA**TTTGA AACGAGATTACAGAGC**ATGCCTACTGCCTCGGA**

KD-02: PSMP-YY1_2 (NM_003403.5; EXON 2 – 781-943, CDS, 844-862)

TGCTGTTGACAGTGAGCGCCCTCCTGATTATTCAGAATAT**TAGTGAAGCCACAGATGTA**ATATTC TGAATAATCAGGAGG**TTGCCTACTGCCTCGGA**

CTRL: PSMP-LUC (FIREFLY LUCIFERASE)

TGCTGTTGACAGTGAGCGCCCGCCTGAAGTCTCTGATTAA**TAGTGAAGCCACAGATGTA**TTAAT CAGAGACTTCAGGCGG**TTGCCTACTGCCTCGGA**

Table S1. JASPAR *in-silico* analysis of putative YY1 transcriptional targets among the main apoptosis regulators.

GENE ALIAS	GENE ID	Relative score	Sequence ID	Start	End	Strand	Predicted sequence
BCL2	BCL2	0,823	chromosome:GRCh38:18:63122746:63322128:-1	308	319	+	CAGTATGGCGAA
BCL2	BCL2	0,819	chromosome:GRCh38:18:63122746:63322128:-1	2689	2700	-	AAAAAAGGCAGC
BCL2	BCL2	0,816	chromosome:GRCh38:18:63122746:63322128:-1	82	93	-	AGAGATGGCTTT
BCL-Xs/XL	BCL2L1	0,900	chromosome:GRCh38:20:31663852:31725989:-1	2692	2703	-	CACAATGGCCGC
BCL-Xs/XL	BCL2L1	0,891	chromosome:GRCh38:20:31663852:31725989:-1	938	949	+	CAAAATGGTGAA
BCL-Xs/XL	BCL2L1	0,848	chromosome:GRCh38:20:31663852:31725989:-1	508	519	-	CGAGATGGAGTC
BCL-Xs/XL	BCL2L1	0,848	chromosome:GRCh38:20:31663852:31725989:-1	1115	1126	-	CGAGATGGAGTC
BCL-Xs/XL	BCL2L1	0,828	chromosome:GRCh38:20:31663852:31725989:-1	1074	1085	+	CAAGATAGCACC
BCL-Xs/XL	BCL2L1	0,823	chromosome:GRCh38:20:31663852:31725989:-1	641	652	+	CAACATGGGGAA
BCL-W	BCL2L2	0,937	chromosome:GRCh38:14:23296790:23312359:1	2102	2113	-	AAAGATGGCTGA
BCL-W	BCL2L2	0,920	chromosome:GRCh38:14:23296790:23312359:1	1831	1842	+	CAACATGGCAGT
BCL-W	BCL2L2	0,808	chromosome:GRCh38:14:23296790:23312359:1	2814	2825	+	CAAAATGAAGGT
BCL-W	BCL2L2	0,800	chromosome:GRCh38:14:23296790:23312359:1	1552	1563	+	CATCATGGTTAC
MCL1	BCL2L3	0,935	chromosome:GRCh38:1:150573951:150581738:-1	996	1007	-	CAACATGGCTGT
MCL1	BCL2L3	0,884	chromosome:GRCh38:1:150573951:150581738:-1	1373	1384	+	AAAAATGGTGTC
MCL1	BCL2L3	0,875	chromosome:GRCh38:1:150573951:150581738:-1	606	617	+	CAACATGGTGAA
MCL1	BCL2L3	0,870	chromosome:GRCh38:1:150573951:150581738:-1	2557	2568	-	CATGATGGCGTC
MCL1	BCL2L3	0,864	chromosome:GRCh38:1:150573951:150581738:-1	386	397	+	CAAAAAGGCTGC
MCL1	BCL2L3	0,841	chromosome:GRCh38:1:150573951:150581738:-1	747	758	-	CACAATGGTGGA
MCL1	BCL2L3	0,826	chromosome:GRCh38:1:150573951:150581738:-1	2535	2546	+	GGAGATGGAAGC
BAX	BCL2L4	0,984	chromosome:GRCh38:19:48952815:48962398:1	1995	2006	-	CAAAATGGCCGC
BAX	BCL2L4	0,887	chromosome:GRCh38:19:48952815:48962398:1	1819	1830	+	AAAAATGGTGCC
BAX	BCL2L4	0,832	chromosome:GRCh38:19:48952815:48962398:1	1093	1104	+	AAATATGGCATA
BAX	BCL2L4	0,828	chromosome:GRCh38:19:48952815:48962398:1	2766	2777	-	CAAAAGGGCCCC
BAX	BCL2L4	0,806	chromosome:GRCh38:19:48952815:48962398:1	984	995	-	CAGGATGGAGTG
BAX	BCL2L4	0,803	chromosome:GRCh38:19:48952815:48962398:1	845	856	-	AAAGATGGGGTT
BAK1	BCL2L7	0,880	chromosome:GRCh38:6:33571947:33582293:-1	958	969	+	AAACATGGCTCA
BAK1	BCL2L7	0,878	chromosome:GRCh38:6:33571947:33582293:-1	467	478	-	TAACATGGCGAA
BAK1	BCL2L7	0,878	chromosome:GRCh38:6:33571947:33582293:-1	2883	2894	-	TAAGATGGCCTT
BAK1	BCL2L7	0,842	chromosome:GRCh38:6:33571947:33582293:-1	387	398	+	CAGGATGGTCTC

BAK1	BCL2L7	0,837	chromosome:GRCh38:6:33571947:33582293:-1	1566	1577	+	CAGAATGGTCTC
BAK1	BCL2L7	0,835	chromosome:GRCh38:6:33571947:33582293:-1	373	384	-	TAACATGGTGAA
BAK1	BCL2L7	0,835	chromosome:GRCh38:6:33571947:33582293:-1	1552	1563	-	TAACATGGTGAA
BAK1	BCL2L7	0,832	chromosome:GRCh38:6:33571947:33582293:-1	432	443	-	CAAAAGGGAGGC
BAK1	BCL2L7	0,819	chromosome:GRCh38:6:33571947:33582293:-1	892	903	-	AAAAAAGGCAGC
BAK1	BCL2L7	0,816	chromosome:GRCh38:6:33571947:33582293:-1	2218	2229	-	GGAGATGGAGGT
BAK1	BCL2L7	0,814	chromosome:GRCh38:6:33571947:33582293:-1	848	859	+	CCAAATGGCTGG
BAK1	BCL2L7	0,808	chromosome:GRCh38:6:33571947:33582293:-1	1374	1385	+	TGAGATGGAGTC
BAK1	BCL2L7	0,804	chromosome:GRCh38:6:33571947:33582293:-1	275	286	-	GAGAATGGCGTG
BAD	BCL2L8	0,955	chromosome:GRCh38:11:64269230:64286704:-1	1223	1234	+	CAAGATGGCCCC
BAD	BCL2L8	0,913	chromosome:GRCh38:11:64269230:64286704:-1	2759	2770	+	AAAAATGGTTGC
BAD	BCL2L8	0,900	chromosome:GRCh38:11:64269230:64286704:-1	2509	2520	-	CATGATGGCTGC
BAD	BCL2L8	0,841	chromosome:GRCh38:11:64269230:64286704:-1	1460	1471	+	CGAGATGGCGCG
BAD	BCL2L8	0,812	chromosome:GRCh38:11:64269230:64286704:-1	1016	1027	+	CAGGAGGGCGGC
BAD	BCL2L8	0,812	chromosome:GRCh38:11:64269230:64286704:-1	2514	2525	+	CATCATGGAGGT
BAD	BCL2L8	0,812	chromosome:GRCh38:11:64269230:64286704:-1	1410	1421	-	TGGGATGGCTGC
BFK	BCL2L15	0,894	chromosome:GRCh38:1:113876216:113889581:-1	867	878	-	CAACATGGCAAA
BFK	BCL2L15	0,885	chromosome:GRCh38:1:113876216:113889581:-1	537	548	-	TAAAATGGCCAA
BFK	BCL2L15	0,867	chromosome:GRCh38:1:113876216:113889581:-1	450	461	-	CAAAATGGTACA
BFK	BCL2L15	0,832	chromosome:GRCh38:1:113876216:113889581:-1	1435	1446	-	CAAAAAGGAGGC
BFK	BCL2L15	0,830	chromosome:GRCh38:1:113876216:113889581:-1	441	452	+	CAAAGTGGCTGT
BFK	BCL2L15	0,801	chromosome:GRCh38:1:113876216:113889581:-1	1161	1172	+	AATGATGGAGTC
BFK	BCL2L15	0,801	chromosome:GRCh38:1:113876216:113889581:-1	73	84	-	CACCATGGCACT
NAIP	BIRC1	0,914	chromosome:GRCh38:5:70967883:71027114:-1	2848	2859	-	TAAAATGGAGGC
NAIP	BIRC1	0,875	chromosome:GRCh38:5:70967883:71027114:-1	1553	1564	+	CAACATGGTGAA
NAIP	BIRC1	0,859	chromosome:GRCh38:5:70967883:71027114:-1	18	29	+	AAAGATGGTTAA
NAIP	BIRC1	0,842	chromosome:GRCh38:5:70967883:71027114:-1	1539	1550	-	CAGGATGGTCTC
NAIP	BIRC1	0,839	chromosome:GRCh38:5:70967883:71027114:-1	2180	2191	+	CAGGATGGGGGC
NAIP	BIRC1	0,835	chromosome:GRCh38:5:70967883:71027114:-1	254	265	+	TAACATGGTGAA
NAIP	BIRC1	0,810	chromosome:GRCh38:5:70967883:71027114:-1	2396	2407	+	CAAGAAGGCGAT
NAIP	BIRC1	0,809	chromosome:GRCh38:5:70967883:71027114:-1	1306	1317	+	AGAAATGGCCTT
NAIP	BIRC1	0,804	chromosome:GRCh38:5:70967883:71027114:-1	1138	1149	-	CCAGATGGCAGG
NAIP	BIRC1	0,803	chromosome:GRCh38:5:70967883:71027114:-1	1949	1960	-	CAATATTGCAGC

NAIP	BIRC1	0,801	chromosome:GRCh38:5:70967883:71027114:-1	1852	1863	+	TAATATGGAGAT
cIAP1	BIRC2	0,850	chromosome:GRCh38:11:102345211:102379270:1	102	113	-	TAAGATGGACAA
cIAP1	BIRC2	0,820	chromosome:GRCh38:11:102345211:102379270:1	1532	1543	-	GAGGATGGAGTC
cIAP2	BIRC3	0,887	chromosome:GRCh38:11:102315450:102340003:1	868	879	+	GAAAATGGCTTA
cIAP2	BIRC3	0,875	chromosome:GRCh38:11:102315450:102340003:1	279	290	-	CAACATGGTGAA
cIAP2	BIRC3	0,862	chromosome:GRCh38:11:102315450:102340003:1	1233	1244	+	AAATATGGCAGT
cIAP2	BIRC3	0,827	chromosome:GRCh38:11:102315450:102340003:1	1446	1457	+	TAAAATGGTAAA
cIAP2	BIRC3	0,814	chromosome:GRCh38:11:102315450:102340003:1	2575	2586	+	CAGCATGGAAAC
XIAP	BIRC4	0,940	chromosome:GRCh38:X:123857724:123914579:1	2191	2202	+	AAAAATGGCGGA
XIAP	BIRC4	0,875	chromosome:GRCh38:X:123857724:123914579:1	823	834	-	CAACATGGTGAA
XIAP	BIRC4	0,854	chromosome:GRCh38:X:123857724:123914579:1	219	230	+	AAAAATGGTTAA
XIAP	BIRC4	0,842	chromosome:GRCh38:X:123857724:123914579:1	529	540	+	CAGGATGGTCTC
XIAP	BIRC4	0,838	chromosome:GRCh38:X:123857724:123914579:1	177	188	+	GGAGATGGTTGC
XIAP	BIRC4	0,827	chromosome:GRCh38:X:123857724:123914579:1	228	239	+	TAAAATGGTAAA
XIAP	BIRC4	0,818	chromosome:GRCh38:X:123857724:123914579:1	118	129	+	CTACATGGCAGA
XIAP	BIRC4	0,804	chromosome:GRCh38:X:123857724:123914579:1	417	428	-	GAGAATGGCGTG
XIAP	BIRC4	0,801	chromosome:GRCh38:X:123857724:123914579:1	1412	1423	-	GAAAATGGGATC
Survivin	BIRC5	0,845	chromosome:GRCh38:17:78212186:78226236:1	770	781	+	AAACATGGTGAA
Survivin	BIRC5	0,845	chromosome:GRCh38:17:78212186:78226236:1	155	166	-	CACCATGGCCTC
Survivin	BIRC5	0,807	chromosome:GRCh38:17:78212186:78226236:1	2491	2502	+	GCAGATGGCCGA
Survivin	BIRC5	0,804	chromosome:GRCh38:17:78212186:78226236:1	2819	2830	+	TAAGATGCCTGA
Survivin	BIRC5	0,804	chromosome:GRCh38:17:78212186:78226236:1	1002	1013	+	AAGAATGGGGGC
Livin	BIRC7	0,878	chromosome:GRCh38:20:63233883:63241095:1	2833	2844	-	CAAGCTGGCGGC
Livin	BIRC7	0,833	chromosome:GRCh38:20:63233883:63241095:1	2428	2439	-	CAAGGTGGCCCC
Livin	BIRC7	0,829	chromosome:GRCh38:20:63233883:63241095:1	1813	1824	-	GATGATGGCTCC
Livin	BIRC7	0,802	chromosome:GRCh38:20:63233883:63241095:1	1881	1892	-	GAGCATGGAGAC
BIK	BIK	0,926	chromosome:GRCh38:22:43108750:43130312:1	2500	2511	+	GAAGATGGCGTC
BIK	BIK	0,871	chromosome:GRCh38:22:43108750:43130312:1	194	205	-	AAAGATGGAGAA
BIK	BIK	0,865	chromosome:GRCh38:22:43108750:43130312:1	597	608	+	CAATATGGCAAA
BIK	BIK	0,838	chromosome:GRCh38:22:43108750:43130312:1	75	86	-	AGAGATGGCTGG
BIK	BIK	0,807	chromosome:GRCh38:22:43108750:43130312:1	379	390	-	CATAATGGGGGC
TP53	TP53	0,888	chromosome:GRCh38:17:7661179:7689550:-1	938	949	+	CAGGATGGCTTC
TP53	TP53	0,843	chromosome:GRCh38:17:7661179:7689550:-1	335	346	+	GAAGATGGGGGA

TP53	TP53	0,828	chromosome:GRCh38:17:7661179 :7689550:-1	1618	1629	+	TAAGATGGTGTG
TP53	TP53	0,823	chromosome:GRCh38:17:7661179 :7689550:-1	1278	1289	-	CAAGATAGAAGC
TP53	TP53	0,808	chromosome:GRCh38:17:7661179 :7689550:-1	2834	2845	-	TAACAGGGCTGC
TP53	TP53	0,806	chromosome:GRCh38:17:7661179 :7689550:-1	2694	2705	-	AAAGATCGCTCC

Table S2. ChIP-Seq YY1-BIRC5 interaction levels in the 11 studies deposited in ENCODE.

YY1-ChIP Signal Strength	Sample Name	Sample Summary	Sample Species	Sample Type	Experiment ID
1000	HepG2	Epithelium, hepatocellular carcinoma	*H sapiens*	Cancer	ENCSR000BNT
1000	K562	Myeloid lineage, myeloblasts, chronic myelogenous leukemia	*H sapiens*	Cancer	ENCSR000BMH
1000	SK-N-SH	Brain, derived from metastatic site: bone marrow, neuroblastoma	*H sapiens*	Cancer	ENCSR000BLZ
734	A549	Epithelium, lung carcinoma	*H sapiens*	Cancer	ENCSR000BPM
319	K562	Myeloid lineage, myeloblasts, chronic myelogenous leukemia	*H sapiens*	Cancer	ENCSR000EWF
279	NT2/D1	Testis; embryonal carcinoma derived from metastatic site: lung	*H sapiens*	Cancer	ENCSR000EXG
1000	GM12878	Lymphoid lineage, B-lymphoblastoid cell line	*H sapiens*	Normal	ENCSR000BNP
947	GM12892	Lymphoid lineage, B-lymphoblastoid cell line	*H sapiens*	Normal	ENCSR000BLT
846	H1-hESC	Pluripotent, embryonic stem cells	*H sapiens*	Normal	ENCSR000BKD
831	GM12891	Lymphoid lineage, B-lymphoblastoid cell line	*H sapiens*	Normal	ENCSR000BKJ
418	Liver	Normal adult liver tissue	*H sapiens*	Normal	ENCSR382MOM

*signal strength score range 0 to 1000

Table S3. Statistics of Figure 2

**YY1- MAS5,0 - u133p2**						
Number of families	1					
Number of comparisons per family	66					
Alpha	0,05					
Tukey's multiple comparisons test	Mean Diff,	95,00% CI of diff,	Significant ?	Summar y	Adjusted P Value	
T- GSE26673 - 16 vs. T- GSE132929 - 290	0,3311	-0,1041 to 0,7663	No	ns	0,3472	A-B
T- GSE26673 - 16 vs. T- GSE10846 - 420	0,5089	0,07725 to 0,9406	Yes	**	0,0066	A-C
T- GSE26673 - 16 vs. T- GSE56315 - 122	1,159	0,7086 to 1,61	Yes	****	<0,0001	A-D
T- GSE26673 - 16 vs. T- GSE87371 - 223	0,3616	-0,07701 to 0,8002	No	ns	0,2282	A-E
T- GSE26673 - 16 vs. T- GSE81183 - 42	0,3447	-0,1532 to 0,8426	No	ns	0,5022	A-F
T- GSE26673 - 16 vs. T- GSE93261 - 149	0,3878	-0,05806 to 0,8336	No	ns	0,1619	A-G
T- GSE26673 - 16 vs. T- GSE16024 - 14	0,7866	0,1664 to 1,407	Yes	**	0,002	A-H
T- GSE26673 - 16 vs. T- GSE93291 -122	0,6979	0,2473 to 1,149	Yes	****	<0,0001	A-I
T- GSE26673 - 16 vs. N- GSE7307 - 504	2,525	2,094 to 2,955	Yes	****	<0,0001	A-J
T- GSE26673 - 16 vs. N- GSE3526 - 353	2,717	2,283 to 3,15	Yes	****	<0,0001	A-K
T- GSE26673 - 16 vs. N- GSE46510 - 154	1,263	0,8181 to 1,708	Yes	****	<0,0001	A-L
T- GSE132929 - 290 vs. T- GSE10846 - 420	0,1778	0,0484 to 0,3072	Yes	***	0,0005	B-C
T- GSE132929 - 290 vs. T- GSE56315 - 122	0,828	0,6452 to 1,011	Yes	****	<0,0001	B-D
T- GSE132929 - 290 vs. T- GSE87371 - 223	0,03047	-0,1205 to 0,1814	No	ns	>0,9999	B-E
T- GSE132929 - 290 vs. T- GSE81183 - 42	0,01357	-0,2662 to 0,2934	No	ns	>0,9999	B-F
T- GSE132929 - 290 vs. T- GSE93261 - 149	0,05665	-0,1142 to 0,2275	No	ns	0,9953	B-G
T- GSE132929 - 290 vs. T- GSE16024 - 14	0,4555	-0,008248 to 0,9192	No	ns	0,0596	B-H
T- GSE132929 - 290 vs. T- GSE93291 -122	0,3668	0,1839 to 0,5497	Yes	****	<0,0001	B-I
T- GSE132929 - 290 vs. N- GSE7307 - 504	2,194	2,069 to 2,318	Yes	****	<0,0001	B-J
T- GSE132929 - 290 vs. N- GSE3526 - 353	2,386	2,251 to 2,52	Yes	****	<0,0001	B-K
T- GSE132929 - 290 vs. N- GSE46510 - 154	0,9321	0,7631 to 1,101	Yes	****	<0,0001	B-L
T- GSE10846 - 420 vs. T- GSE56315 - 122	0,6502	0,4759 to 0,8245	Yes	****	<0,0001	C-D
T- GSE10846 - 420 vs. T- GSE87371 - 223	-0,1473	-0,2877 to -0,006903	Yes	*	0,03	C-E
T- GSE10846 - 420 vs. T- GSE81183 - 42	-0,1642	-0,4385 to 0,11	No	ns	0,7215	C-F
T- GSE10846 - 420 vs. T- GSE93261 - 149	-0,1211	-0,2827 to 0,04045	No	ns	0,3711	C-G
T- GSE10846 - 420 vs. T- GSE16024 - 14	0,2777	-0,1827 to 0,7381	No	ns	0,7119	C-H
T- GSE10846 - 420 vs. T- GSE93291 -122	0,189	0,01472 to 0,3633	Yes	*	0,0203	C-I
T- GSE10846 - 420 vs. N- GSE7307 - 504	2,016	1,904 to 2,128	Yes	****	<0,0001	C-J
T- GSE10846 - 420 vs. N- GSE3526 - 353	2,208	2,085 to 2,33	Yes	****	<0,0001	C-K
T- GSE10846 - 420 vs. N- GSE46510 - 154	0,7543	0,5947 to 0,914	Yes	****	<0,0001	C-L
T- GSE56315 - 122 vs. T- GSE87371 - 223	-0,7976	-0,9884 to -0,6067	Yes	****	<0,0001	D-E
T- GSE56315 - 122 vs. T- GSE81183 - 42	-0,8145	-1,118 to -0,5113	Yes	****	<0,0001	D-F
T- GSE56315 - 122 vs. T- GSE93261 - 149	-0,7714	-0,9783 to -0,5645	Yes	****	<0,0001	D-G
T- GSE56315 - 122 vs. T- GSE16024 - 14	-0,3726	-0,8508 to 0,1057	No	ns	0,3102	D-H
T- GSE56315 - 122 vs. T- GSE93291 -122	-0,4612	-0,6782 to -0,2442	Yes	****	<0,0001	D-I
T- GSE56315 - 122 vs. N- GSE7307 - 504	1,366	1,195 to 1,537	Yes	****	<0,0001	D-J
T- GSE56315 - 122 vs. N- GSE3526 - 353	1,557	1,38 to 1,735	Yes	****	<0,0001	D-K
T- GSE56315 - 122 vs. N- GSE46510 - 154	0,1041	-0,1013 to 0,3095	No	ns	0,8871	D-L
T- GSE87371 - 223 vs. T- GSE81183 - 42	-0,0169	-0,302 to 0,2682	No	ns	>0,9999	E-F

T- GSE87371 - 223 vs. T- GSE93261 - 149	0,02618	-0,1531 to 0,2055	No	ns	>0,9999	E-G
T- GSE87371 - 223 vs. T- GSE16024 - 14	0,425	-0,04191 to 0,8919	No	ns	0,1157	E-H
T- GSE87371 - 223 vs. T- GSE93291 -122	0,3363	0,1455 to 0,5272	Yes	****	<0,0001	E-I
T- GSE87371 - 223 vs. N- GSE7307 - 504	2,163	2,027 to 2,299	Yes	****	<0,0001	E-J
T- GSE87371 - 223 vs. N- GSE3526 - 353	2,355	2,21 to 2,5	Yes	****	<0,0001	E-K
T- GSE87371 - 223 vs. N- GSE46510 - 154	0,9016	0,7241 to 1,079	Yes	****	<0,0001	E-L
T- GSE81183 - 42 vs. T- GSE93261 - 149	0,04307	-0,253 to 0,3391	No	ns	>0,9999	F-G
T- GSE81183 - 42 vs. T- GSE16024 - 14	0,4419	-0,08108 to 0,9649	No	ns	0,1962	F-H
T- GSE81183 - 42 vs. T- GSE93291 -122	0,3532	0,05005 to 0,6564	Yes	**	0,0078	F-I
T- GSE81183 - 42 vs. N- GSE7307 - 504	2,18	1,908 to 2,452	Yes	****	<0,0001	F-J
T- GSE81183 - 42 vs. N- GSE3526 - 353	2,372	2,095 to 2,649	Yes	****	<0,0001	F-K
T- GSE81183 - 42 vs. N- GSE46510 - 154	0,9185	0,6235 to 1,214	Yes	****	<0,0001	F-L
T- GSE93261 - 149 vs. T- GSE16024 - 14	0,3988	-0,07489 to 0,8726	No	ns	0,2008	G-H
T- GSE93261 - 149 vs. T- GSE93291 -122	0,3102	0,1032 to 0,5171	Yes	****	<0,0001	G-I
T- GSE93261 - 149 vs. N- GSE7307 - 504	2,137	1,979 to 2,295	Yes	****	<0,0001	G-J
T- GSE93261 - 149 vs. N- GSE3526 - 353	2,329	2,163 to 2,494	Yes	****	<0,0001	G-K
T- GSE93261 - 149 vs. N- GSE46510 - 154	0,8755	0,6807 to 1,07	Yes	****	<0,0001	G-L
T- GSE16024 - 14 vs. T- GSE93291 -122	-0,08868	-0,5669 to 0,3895	No	ns	>0,9999	H-I
T- GSE16024 - 14 vs. N- GSE7307 - 504	1,738	1,279 to 2,197	Yes	****	<0,0001	H-J
T- GSE16024 - 14 vs. N- GSE3526 - 353	1,93	1,468 to 2,392	Yes	****	<0,0001	H-K
T- GSE16024 - 14 vs. N- GSE46510 - 154	0,4766	0,003562 to 0,9497	Yes	*	0,0463	H-L
T- GSE93291 -122 vs. N- GSE7307 - 504	1,827	1,656 to 1,998	Yes	****	<0,0001	I-J
T- GSE93291 -122 vs. N- GSE3526 - 353	2,019	1,841 to 2,197	Yes	****	<0,0001	I-K
T- GSE93291 -122 vs. N- GSE46510 - 154	0,5653	0,3599 to 0,7707	Yes	****	<0,0001	I-L
N- GSE7307 - 504 vs. N- GSE3526 - 353	0,192	0,07434 to 0,3096	Yes	****	<0,0001	J-K
N- GSE7307 - 504 vs. N- GSE46510 - 154	-1,261	-1,417 to -1,105	Yes	****	<0,0001	J-L
N- GSE3526 - 353 vs. N- GSE46510 - 154	-1,453	-1,617 to -1,29	Yes	****	<0,0001	K-L

BIRC5 - MAS5,0 - u133p2

Number of families	1
Number of comparisons per family	66
Alpha	0,05

Tukey's multiple comparisons test	Mean Diff,	95,00% CI of diff,	Significant ?	Summary	Adjusted P Value	
T- GSE26673 - 16 vs. T- GSE132929 - 290	1,328	0,1828 to 2,473	Yes	**	0,0084	A-B
T- GSE26673 - 16 vs. T- GSE10846 - 420	0,8255	-0,3103 to 1,961	No	ns	0,4217	A-C
T- GSE26673 - 16 vs. T- GSE56315 - 122	1,506	0,3205 to 2,692	Yes	**	0,002	A-D
T- GSE26673 - 16 vs. T- GSE87371 - 223	1,628	0,4738 to 2,782	Yes	***	0,0003	A-E
T- GSE26673 - 16 vs. T- GSE81183 - 42	0,5335	-0,7765 to 1,843	No	ns	0,9749	A-F
T- GSE26673 - 16 vs. T- GSE93261 - 149	2,536	1,362 to 3,709	Yes	****	<0,0001	A-G
T- GSE26673 - 16 vs. T- GSE16024 - 14	2,369	0,7374 to 4,001	Yes	***	0,0001	A-H
T- GSE26673 - 16 vs. T- GSE93291 -122	2,431	1,245 to 3,616	Yes	****	<0,0001	A-I
T- GSE26673 - 16 vs. N- GSE7307 - 504	5,745	4,613 to 6,878	Yes	****	<0,0001	A-J
T- GSE26673 - 16 vs. N- GSE3526 - 353	5,97	4,83 to 7,11	Yes	****	<0,0001	A-K
T- GSE26673 - 16 vs. N- GSE46510 - 154	6,117	4,946 to 7,288	Yes	****	<0,0001	A-L
T- GSE132929 - 290 vs. T- GSE10846 - 420	-0,5024	-0,8428 to -0,1619	Yes	****	<0,0001	B-C

T- GSE132929 - 290 vs. T- GSE56315 - 122	0,1783	-0,3029 to 0,6594	No	ns	0,9881	B-D
T- GSE132929 - 290 vs. T- GSE87371 - 223	0,3	-0,09713 to 0,6971	No	ns	0,3587	B-E
T- GSE132929 - 290 vs. T- GSE81183 - 42	-0,7944	-1,531 to -0,05822	Yes	*	0,0216	B-F
T- GSE132929 - 290 vs. T- GSE93261 - 149	1,208	0,7583 to 1,657	Yes	****	<0,0001	B-G
T- GSE132929 - 290 vs. T- GSE16024 - 14	1,041	-0,1788 to 2,261	No	ns	0,1839	B-H
T- GSE132929 - 290 vs. T- GSE93291 -122	1,103	0,6215 to 1,584	Yes	****	<0,0001	B-I
T- GSE132929 - 290 vs. N- GSE7307 - 504	4,417	4,089 to 4,746	Yes	****	<0,0001	B-J
T- GSE132929 - 290 vs. N- GSE3526 - 353	4,642	4,289 to 4,996	Yes	****	<0,0001	B-K
T- GSE132929 - 290 vs. N- GSE46510 - 154	4,789	4,345 to 5,234	Yes	****	<0,0001	B-L
T- GSE10846 - 420 vs. T- GSE56315 - 122	0,6806	0,222 to 1,139	Yes	****	<0,0001	C-D
T- GSE10846 - 420 vs. T- GSE87371 - 223	0,8024	0,4329 to 1,172	Yes	****	<0,0001	C-E
T- GSE10846 - 420 vs. T- GSE81183 - 42	-0,292	-1,014 to 0,4296	No	ns	0,9761	C-F
T- GSE10846 - 420 vs. T- GSE93261 - 149	1,71	1,285 to 2,135	Yes	****	<0,0001	C-G
T- GSE10846 - 420 vs. T- GSE16024 - 14	1,544	0,3323 to 2,755	Yes	**	0,0019	C-H
T- GSE10846 - 420 vs. T- GSE93291 -122	1,605	1,146 to 2,064	Yes	****	<0,0001	C-I
T- GSE10846 - 420 vs. N- GSE7307 - 504	4,92	4,625 to 5,214	Yes	****	<0,0001	C-J
T- GSE10846 - 420 vs. N- GSE3526 - 353	5,145	4,823 to 5,467	Yes	****	<0,0001	C-K
T- GSE10846 - 420 vs. N- GSE46510 - 154	5,292	4,872 to 5,712	Yes	****	<0,0001	C-L
T- GSE56315 - 122 vs. T- GSE87371 - 223	0,1218	-0,3804 to 0,6239	No	ns	0,9997	D-E
T- GSE56315 - 122 vs. T- GSE81183 - 42	-0,9726	-1,77 to -0,1749	Yes	**	0,0039	D-F
T- GSE56315 - 122 vs. T- GSE93261 - 149	1,029	0,485 to 1,574	Yes	****	<0,0001	D-G
T- GSE56315 - 122 vs. T- GSE16024 - 14	0,8631	-0,3951 to 2,121	No	ns	0,5175	D-H
T- GSE56315 - 122 vs. T- GSE93291 -122	0,9244	0,3535 to 1,495	Yes	****	<0,0001	D-I
T- GSE56315 - 122 vs. N- GSE7307 - 504	4,239	3,789 to 4,689	Yes	****	<0,0001	D-J
T- GSE56315 - 122 vs. N- GSE3526 - 353	4,464	3,996 to 4,932	Yes	****	<0,0001	D-K
T- GSE56315 - 122 vs. N- GSE46510 - 154	4,611	4,071 to 5,152	Yes	****	<0,0001	D-L
T- GSE87371 - 223 vs. T- GSE81183 - 42	-1,094	-1,844 to -0,3444	Yes	***	0,0001	E-F
T- GSE87371 - 223 vs. T- GSE93261 - 149	0,9077	0,4359 to 1,379	Yes	****	<0,0001	E-G
T- GSE87371 - 223 vs. T- GSE16024 - 14	0,7413	-0,4872 to 1,97	No	ns	0,7113	E-H
T- GSE87371 - 223 vs. T- GSE93291 -122	0,8027	0,3006 to 1,305	Yes	****	<0,0001	E-I
T- GSE87371 - 223 vs. N- GSE7307 - 504	4,117	3,759 to 4,476	Yes	****	<0,0001	E-J
T- GSE87371 - 223 vs. N- GSE3526 - 353	4,342	3,961 to 4,724	Yes	****	<0,0001	E-K
T- GSE87371 - 223 vs. N- GSE46510 - 154	4,489	4,022 to 4,957	Yes	****	<0,0001	E-L
T- GSE81183 - 42 vs. T- GSE93261 - 149	2,002	1,223 to 2,781	Yes	****	<0,0001	F-G
T- GSE81183 - 42 vs. T- GSE16024 - 14	1,836	0,4597 to 3,212	Yes	***	0,0008	F-H
T- GSE81183 - 42 vs. T- GSE93291 -122	1,897	1,099 to 2,695	Yes	****	<0,0001	F-I
T- GSE81183 - 42 vs. N- GSE7307 - 504	5,212	4,496 to 5,928	Yes	****	<0,0001	F-J
T- GSE81183 - 42 vs. N- GSE3526 - 353	5,437	4,709 to 6,164	Yes	****	<0,0001	F-K
T- GSE81183 - 42 vs. N- GSE46510 - 154	5,584	4,808 to 6,36	Yes	****	<0,0001	F-L
T- GSE93261 - 149 vs. T- GSE16024 - 14	-0,1664	-1,413 to 1,08	No	ns	>0,9999	G-H
T- GSE93261 - 149 vs. T- GSE93291 -122	-0,105	-0,6494 to 0,4394	No	ns	>0,9999	G-I
T- GSE93261 - 149 vs. N- GSE7307 - 504	3,21	2,794 to 3,626	Yes	****	<0,0001	G-J
T- GSE93261 - 149 vs. N- GSE3526 - 353	3,435	2,999 to 3,87	Yes	****	<0,0001	G-K
T- GSE93261 - 149 vs. N- GSE46510 - 154	3,582	3,069 to 4,094	Yes	****	<0,0001	G-L
T- GSE16024 - 14 vs. T- GSE93291 -122	0,06135	-1,197 to 1,32	No	ns	>0,9999	H-I
T- GSE16024 - 14 vs. N- GSE7307 - 504	3,376	2,168 to 4,584	Yes	****	<0,0001	H-J
T- GSE16024 - 14 vs. N- GSE3526 - 353	3,601	2,386 to 4,816	Yes	****	<0,0001	H-K

T- GSE16024 - 14 vs. N- GSE46510 - 154	3,748	2,503 to 4,993	Yes	****	<0,0001	H-L
T- GSE93291 -122 vs. N- GSE7307 - 504	3,315	2,865 to 3,765	Yes	****	<0,0001	I-J
T- GSE93291 -122 vs. N- GSE3526 - 353	3,54	3,071 to 4,008	Yes	****	<0,0001	I-K
T- GSE93291 -122 vs. N- GSE46510 - 154	3,687	3,146 to 4,227	Yes	****	<0,0001	I-L
N- GSE7307 - 504 vs. N- GSE3526 - 353	0,2248	-0,08469 to 0,5343	No	ns	0,4228	J-K
N- GSE7307 - 504 vs. N- GSE46510 - 154	0,3718	-0,03872 to 0,7824	No	ns	0,1203	J-L
N- GSE3526 - 353 vs. N- GSE46510 - 154	0,1471	-0,2836 to 0,5777	No	ns	0,994	K-L

YY1- MAS5,0 - u133a

Number of families	1					
Number of comparisons per family	10					
Alpha	0,05					
Tukey's multiple comparisons test	Mean Diff,	95,00% CI of diff,	Significant ?	Summary	Adjusted P Value	
T- GSE4475 - 215 vs. T- GSE10172 - 36	0,1021	-0,1452 to 0,3493	No	ns	0,791	A-B
T- GSE4475 - 215 vs. T- GSE57611 - 148	0,1434	-0,003301 to 0,29	No	ns	0,059	A-C
T- GSE4475 - 215 vs. N- GSE1133 - 158	1,651	1,507 to 1,795	Yes	****	<0,0001	A-D
T- GSE4475 - 215 vs. N- GSE3846 - 108	1,191	1,03 to 1,353	Yes	****	<0,0001	A-E
T- GSE10172 - 36 vs. T- GSE57611 - 148	0,04126	-0,2139 to 0,2964	No	ns	0,9921	B-C
T- GSE10172 - 36 vs. N- GSE1133 - 158	1,549	1,295 to 1,802	Yes	****	<0,0001	B-D
T- GSE10172 - 36 vs. N- GSE3846 - 108	1,089	0,8251 to 1,354	Yes	****	<0,0001	B-E
T- GSE57611 - 148 vs. N- GSE1133 - 158	1,508	1,35 to 1,665	Yes	****	<0,0001	C-D
T- GSE57611 - 148 vs. N- GSE3846 - 108	1,048	0,8743 to 1,222	Yes	****	<0,0001	C-E
N- GSE1133 - 158 vs. N- GSE3846 - 108	-0,4594	-0,6309 to -0,288	Yes	****	<0,0001	D-E

BIRC5 - MAS5,0 - u133a

Number of families	1					
Number of comparisons per family	10					
Alpha	0,05					
Tukey's multiple comparisons test	Mean Diff,	95,00% CI of diff,	Significant ?	Summary	Adjusted P Value	
T- GSE4475 - 215 vs. T- GSE10172 - 36	-0,3584	-0,9401 to 0,2233	No	ns	0,4438	A-B
T- GSE4475 - 215 vs. T- GSE57611 - 148	0,2598	-0,08518 to 0,6049	No	ns	0,2389	A-C
T- GSE4475 - 215 vs. N- GSE1133 - 158	2,354	2,015 to 2,692	Yes	****	<0,0001	A-D
T- GSE4475 - 215 vs. N- GSE3846 - 108	3,158	2,777 to 3,539	Yes	****	<0,0001	A-E
T- GSE10172 - 36 vs. T- GSE57611 - 148	0,6182	0,01792 to 1,219	Yes	*	0,0399	B-C
T- GSE10172 - 36 vs. N- GSE1133 - 158	2,712	2,116 to 3,309	Yes	****	<0,0001	B-D
T- GSE10172 - 36 vs. N- GSE3846 - 108	3,516	2,895 to 4,138	Yes	****	<0,0001	B-E
T- GSE57611 - 148 vs. N- GSE1133 - 158	2,094	1,725 to 2,464	Yes	****	<0,0001	C-D
T- GSE57611 - 148 vs. N- GSE3846 - 108	2,898	2,489 to 3,307	Yes	****	<0,0001	C-E
N- GSE1133 - 158 vs. N- GSE3846 - 108	0,8039	0,4006 to 1,207	Yes	****	<0,0001	D-E

Table S4. Contingency tables relative to the Fisher's and ROC analyses

**Hummel (GSE4475)**			
Data analyzed	non-MBL	MBL	Total
YY1 High	80	37	117
YY1 Low	91	7	98
Total	171	44	215
Data analyzed	non-MBL	MBL	Total
BIRC5 High	63	41	104
BIRC5 Low	108	3	111
Total	171	44	215
**Siebert (GSE10172)**			
Data analyzed	non-BL	BL	Total
YY1 High	5	16	21
YY1 Low	11	4	15
Total	16	20	36
Data analyzed	non-BL	BL	Total
BIRC5 High	4	14	18
BIRC5 Low	12	6	18
Total	16	20	36
**Green (GSE132929)**			
Data analyzed	Indolent	Aggressive	Total
YY1 High	55	102	157
YY1 Low	76	56	132
Total	131	158	289
Data analyzed	Indolent	Aggressive	Total
BIRC5 High	29	126	155
BIRC5 Low	102	32	134
Total	131	158	289
